# Indirect Access to Carbene Adducts of Bismuth- and Antimony-Substituted Phosphaketene and Their Unusual Thermal Transformation to Dipnictines and [(NHC)_2_OCP][OCP]

**DOI:** 10.1021/acs.inorgchem.0c03683

**Published:** 2021-03-09

**Authors:** Jacob E. Walley, Levi S. Warring, Erik Kertész, Guocang Wang, Diane A. Dickie, Zoltán Benkő, Robert J. Gilliard

**Affiliations:** † Department of Chemistry, University of Virginia, 409 McCormick Road, P.O. Box 400319, Charlottesville, Virginia 22903, United States; ‡ Department of Inorganic and Analytical Chemistry, Budapest University of Technology and Economics, Szent Gellért tér 4, H-1111 Budapest, Hungary

## Abstract

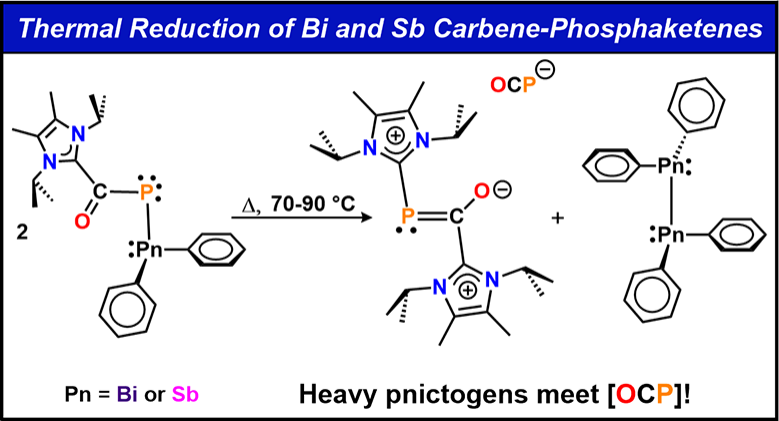

The synthesis and thermal redox chemistry of the first antimony (Sb)– and bismuth (Bi)–phosphaketene adducts are described. When diphenylpnictogen chloride [Ph_2_PnCl (Pn = Sb or Bi)] is reacted with sodium 2-phosphaethynolate [Na[OCP]·(dioxane)_*x*_], tetraphenyldipnictogen (Ph_2_Pn–PnPh_2_) compounds are produced, and an insoluble precipitate forms from solution. In contrast, when the *N*-heterocyclic carbene adduct (NHC)–PnPh_2_Cl is combined with [Na[OCP]·(dioxane)_*x*_], Sb– and Bi–phosphaketene complexes are isolated. Thus, NHC serves as an essential mediator for the reaction. Immediately after the formation of an intermediary pnictogen–phosphaketene NHC adduct [NHC–PnPh_2_(PCO)], the NHC ligand transfers from the Pn center to the phosphaketene carbon atom, forming NHC–C(O)P-PnPh_2_ [Pn = Sb (**3**) or Bi (**4**)]. In the solid state, **3** and **4** are dimeric with short intermolecular Pn–Pn interactions. When compounds **3** and **4** are heated in THF at 90 and 70 °C, respectively, the pnictogen center Pn^III^ is thermally reduced to Pn^II^ to form tetraphenyldipnictines (Ph_2_Pn–PnPh_2_) and an unusual *bis*-carbene-supported OCP salt, [(NHC)_2_OCP][OCP] (**5**). The formation of compound **5** and Ph_2_Pn–PnPh_2_ from **3** or **4** is unique in comparison to the known thermal reactivity for group 14 carbene–phosphaketene complexes, further highlighting the diverse reactivity of [OCP]^−^ with main-group elements. All new compounds have been fully characterized by single-crystal X-ray diffraction, multinuclear NMR spectroscopy (^1^H, ^13^C, and ^31^P), infrared spectroscopy, and elemental analysis (**1**, **2**, and **5**). The electronic structure of **5** and the mechanism of formation were investigated using density functional theory (DFT).

## Abstract 2

An *N*-heterocyclic carbene (NHC) was used to support the otherwise unstable Ph_2_Sb—P=C=O and Ph_2_Bi—P=C=O moieties. Exploration of the thermal chemistry of these NHC−phosphaketene adducts reveals the formation of the salt [NHC_2_OCP][OCP]. This present work demonstrates the thermal chemistry of the 2-phospaethynolate anion with heavier pnictogens (Sb and Bi).

## Introduction

Due to their unique electron distribution, heteroketenes show versatile and fascinating chemistry. Phosphorus-containing members of this family are phosphaketenes, R—P=C=O.^1^ Although the first stable phosphaketene was reported nearly four decades ago,^2^ the synthetic chemistry was experimentally challenging, and various products were thermally unstable. However, in the past decade, simple synthetic routes toward such compounds have emerged, which has resulted in the rapid development of the field. The utilization of the 2-phosphaethynolate anion,^3^ [OCP]^−^, as a synthon has proved to be an effective way to access phosphaketenes via nucleophilic substitution.^4^ However, these synthetic processes are not always straightforward, and phosphaketene stability and reactivity may be hampered by a number of complications. The most important of these are summarized as follows. (*i*) Dimer formation: the P=C bond of phosphaketenes is prone to cycloaddition, which results in 4-membered rings; however, this process can be minimized with the incorporation of bulky substituents, or with heteroatoms.^5^ (*ii*) Formation of constitutional isomers: due to its ambident reactivity, the [OCP]^−^ anion may bind through the P or the O center. Highly oxophilic species, such as s-^3c,6^ or f-block elements,^7^ favor the oxyphosphaalkyne isomer, while soft Lewis acidic elements, for example, the heavy group 14 elements (Ge, Sn, Pb),^4b,8^ and Ga^4c,9^ favor the phosphaketene isomer. Both O- and P-bound isomers are known for B^4a,10^ and Si,^4b^ illustrating the ambident nature of the [OCP]^−^ anion. (*iii*) Redox chemistry: the OCP anion is prone to oxidation by many metals due to its reductive nature and the electrophilic character of the metals.^3a,11^ While point (*i*) can be circumvented using sterically demanding substituents to stabilize the phosphaketene, points (*ii*) and (*iii*) are more challenging to avoid because the inherent electrophilic properties of the main-group elements differ widely across the periodic table. Nevertheless, phosphaketene isomers R—P=C=O are usually more stable than their oxyphosphaalkyne R—O—C≡P analogues; thus (*ii*) is a less common problem in synthetic routes. In this Article, we aim to offer a solution to the problem described in point (*iii*). Since the heavier pnictogens are easily reduced, neutral donor ligands such as *N*-heterocyclic carbenes (NHC) can be employed to stabilize the phosphaketene motif, thereby preventing reduction at the pnictogen center.

**Figure 1:**
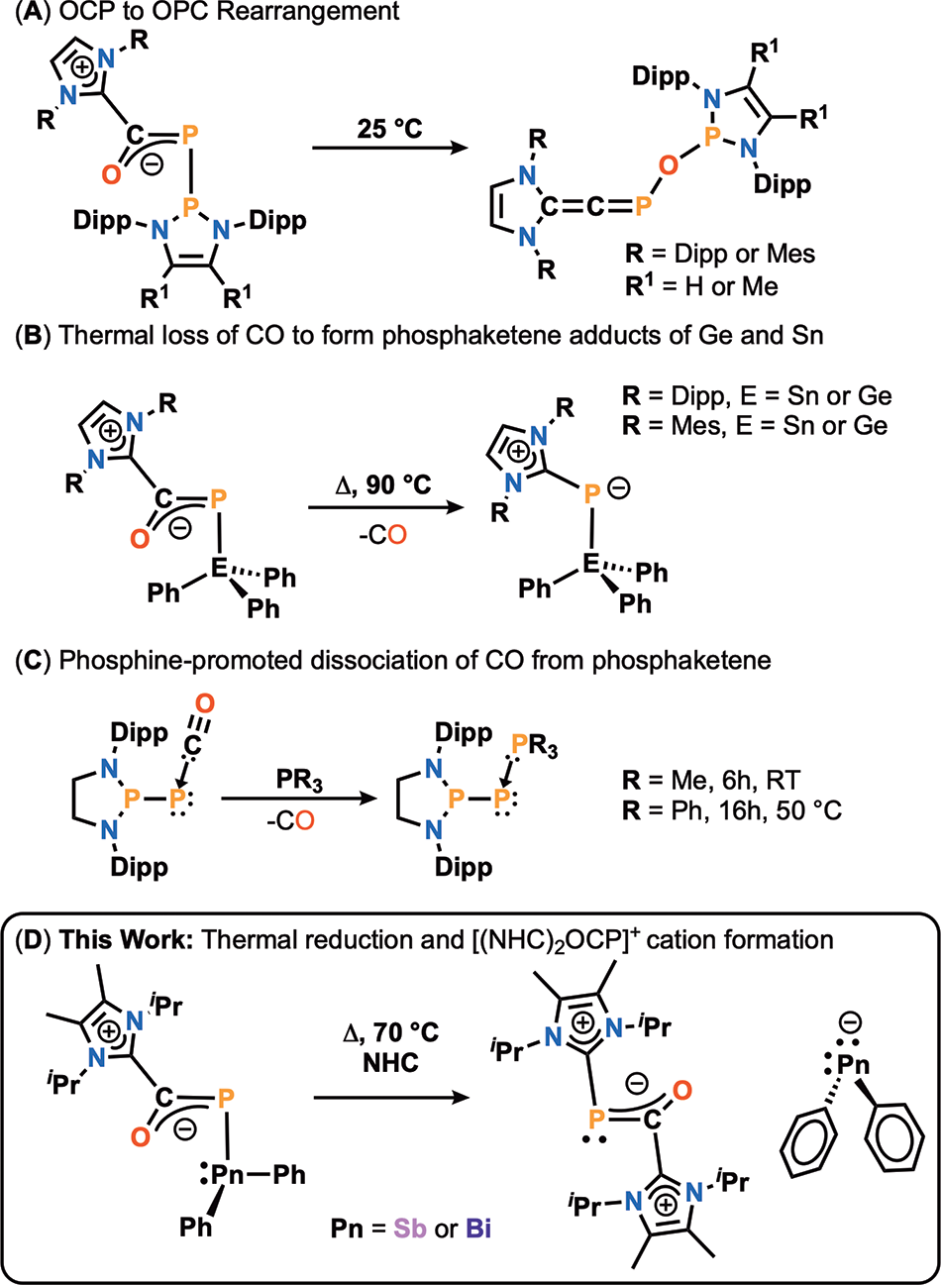
(A) OCP to OPC rearrangement; (B) thermal loss of CO from NHC−phosphaketene adducts of triphenyl-germanium or -tin to form NHC−phosphinidenes; (C) phosphine-promoted CO dissociation; (D) This work: thermal reduction involving Sb and Bi phosphaketene.

Phosphaketenes show rich and often unprecedented chemistry. In recent years, the phosphanyl- and tetrel-substituted phosphaketenes have attracted special interest. Bertrand, Su, and Grützmacher discovered a unique reaction where OCP rearranges to OPC when an *N*-heterocyclic phosphane (NHP)–phosphaketene adduct is reacted with NHC (Figure 1A).^4e^ Nucleophilic attack on the OCP carbon atom by the NHC results in a zwitterionic intermediate, which is followed by migration of the NHP unit to oxygen. Grützmacher et al. showed that the CO unit of the phosphaketene can be substituted by a carbene, demonstrating similar phosphaketene reactivity with NHCs (Figure 1B).^4d^ Addition of NHC to a triphenylgermanium– or tin–phosphaketene led to the formation of NHC–phosphaketene adducts. When heated, the NHC transfers to phosphorus to release CO, thereby forming NHC–phosphinidene germanium and tin complexes. Similarly, the C≡O unit of a phosphaketene can be exchanged by another donor. Bertrand observed loss of CO from NHP–phosphaketenes when a Lewis basic phosphine was introduced with moderate heating (Figure 1C).^12^ It is noteworthy that the loss of CO from P–CO-containing molecules has been explored computationally.^13^ The reaction proceeds via an associative mechanism, whereby the phosphine binds to the −PCO unit first, followed by loss of CO.

Within the realm of main-group elements, the reactivity of Na[OCP] has been established for group 2,^6a,6b^ group 13,^4c,9,10^ group 14,^4b,4e,8,14^ and group 15.^4e,15^ For the lattermost, these examples are limited to phosphorus and arscenic, with no current reports describing reactions of Na[OCP] with the heavier pnictogens (Sb and Bi). Nevertheless, the chemistry of the heavier two Pn elements (Sb, Bi) has seen a substantial increase in interest recently as novel bonding motifs, and new applications in catalysis continue to be discovered.^16^

Herein, we report the first reactions of Na[OCP] with antimony and bismuth compounds, [NHC–Sb(Ph)_2_Cl]_2_ (**1**) and [NHC–Bi(Ph)_2_Cl]_2_ (**2**). [NHC–PnPh_2_Cl]_2_ was combined with [Na[OCP]·(dioxane)_*x*_] to afford Sb– and Bi–phosphaketene complexes (**3** and **4**, respectively). Notably, compounds **3** and **4** were susceptible to a thermal reduction process where the Pn^III^ center is reduced to Pn^II^ to form either tetraphenyldistibine or tetraphenyldibismuthine and the [(NHC)_2_OCP][OCP] salt (**5**). Compound **5** is a unique example of a salt with an [OCP] moiety imbodying both the cation and the anion. DFT calculations demonstrate that the formation of the cationic unit in **5** occurs in a mechanistic step where nucleophilic attack of a dissociated NHC on one unit of **4** leads to the loss of [Ph_2_Bi]^−^ (Figure 1D).

## Results and Discussion

**Scheme 1:**
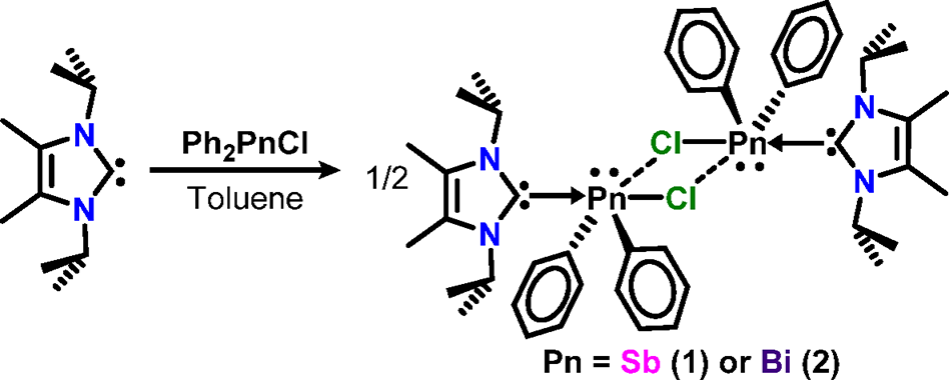
Synthesis of Diphenylpnictogen Halide *N*-Heterocyclic Carbene Complexes

We initially performed the reaction of Na[OCP] with Ph_2_PnCl (Pn = Sb or Bi) and observed the formation of tetraphenyldipnictine and an insoluble unidentified precipitate. Extending the scope of this reaction, NHC ligand 4,5-dimethyl-1,3-diisopropylimidazolin-2-ylidene was reacted with diphenylantimony chloride (Ph_2_SbCl) or diphenylbismuth chloride (Ph_2_BiCl) in THF for 1 h at room temperature (Scheme 1). Compounds **1** (Sb) and **2** (Bi) were obtained as white solids in 94% and 85% yield, respectively. The ^1^H NMR spectrum of **1** in C_6_D_6_ shows a broad heptet at 4.69 ppm, attributed to the NHC methine proton. This is shifted downfield from the methine of the NHC ligand (3.96 ppm). Due to poor solubility in C_6_D_6_, the ^1^H NMR spectrum of compound **2** was recorded in THF-*d*_8_, which showed a broadened heptet at 4.51 ppm, attributed to the methine protons of coordinated NHC.

**Figure 2:**
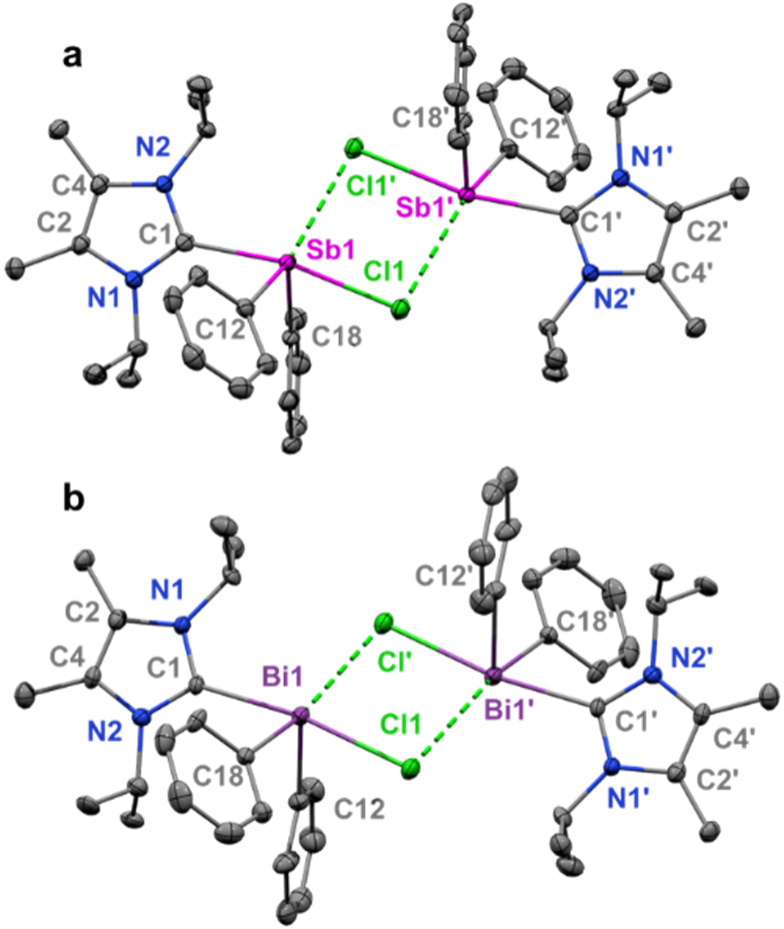
Molecular structure of **1** (a): Thermal ellipsoids at 50% probability; H atoms omitted for clarity. Selected bond distances (Å) and angles (deg): Sb1–C1 2.356(3); Sb1–Cl1 2.8006(8); Sb1–Cl1′ 3.9544(10); Sb1–C18 2.168(3); Sb1–C12 2.171(4). C18–Sb1–C12 102.19(14); C18–Sb1–C1 87.58(12); C12–Sb1–C1 86.33(12); C18–Sb1–Cl1 87.00(8); C12–Sb1–Cl1 85.24(9); C1–Sb1–Cl1 168.80(9). Molecular structure of **2** (b): Thermal ellipsoids at 50% probability; H atoms were omitted for clarity. Selected bond distances (Å) and angles (deg): Bi1–C1 2.489(6); Bi1–Cl1 2.8696(16); Bi1–Cl1′ 3.7211(17); Bi1–C18 2.257(6); Bi1–C12 2.267(6). C18–Bi1–C12 99.0(2); C18–Bi1–C1 86.3(2); C12–Bi1–C1 88.1(2); C18–Bi1–Cl1 86.26(16); C12–Bi1–Cl1 88.26(16); C1–Bi1–Cl1 171.05(15).

Colorless crystals suitable for X-ray diffraction of both **1** and **2** were obtained from toluene/hexane (10:1) mixtures at −37 °C. The molecular structures of compounds **1** and **2** are dimeric with distorted square pyramidal geometry around the metal center (Figure 2). The C1–Sb1 bond distance in compound **1** [2.356(3) Å] is outside the range of other ^NHC^C–Sb bonds (2.144–2.268 Å);^16o,16q,16r,17^ likewise, the C1–Bi1 bond in compound **2** [2.489(6)] is slightly longer than the known range for ^NHC^C–Bi bonds (2.339–2.428 Å).^17b,18^ The Pn–Cl bond lengths in **1** (2.8006(8) Å) and **2** (2.8696(16) Å) are also significantly longer than those in reported complexes containing Sb–Cl (2.332–2.402 Å)^17a−17c^ and Bi–Cl (2.437–2.705 Å)^18a,18b^ bonds. The longer ^NHC^C–Pn bonds results from the weak Lewis acidity of Ph_2_PnCl compared to PhBiCl_2_ and BiCl_3_. The intermolecular Pn–Cl distances in **1** [3.9544(10) Å] are longer than those in **2** [3.7211(17) Å], which is due to the pronounced Lewis acidity at the Bi center.

**Scheme 2:**
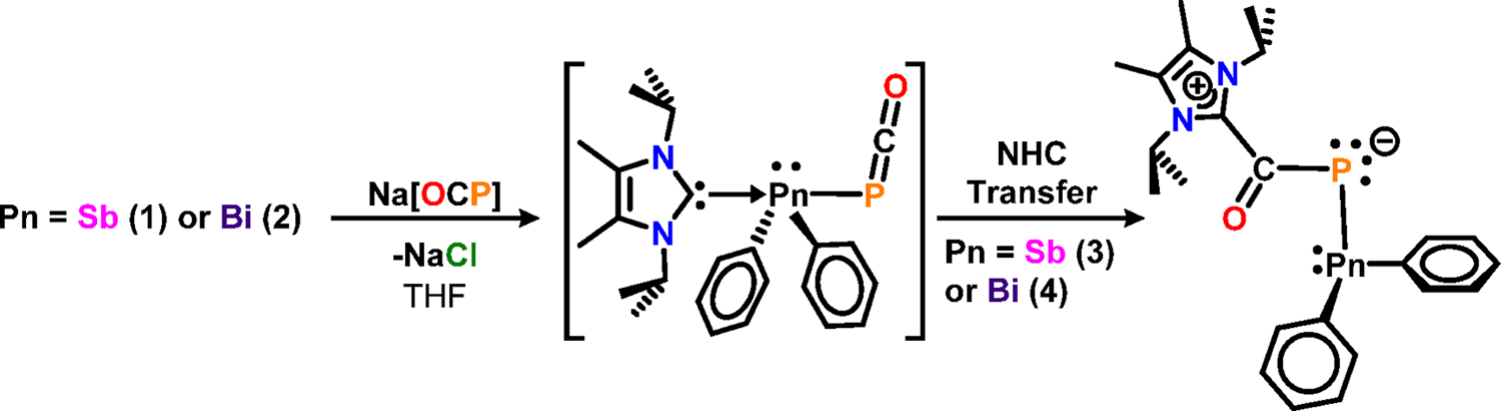
Synthesis of Antimony– and Bismuth–Phosphaketene Adducts

For both compounds **1** and **2**, we hypothesized that a combination of electronic stabilization from the coordinated NHC and steric protection from the two phenyl groups may stabilize their OCP adducts. Based on the reactivity known for [OCP]^−^ with other main group elements,^3a^ we predicted the formation of a pnictogen–phosphaketene adduct, Pn–PCO. Therefore, we reacted compounds **1** and **2** with Na[OCP]·(dioxane)_*x*_ at −37 °C in THF (Scheme 2). The ^31^P NMR spectra of the isolated complexes revealed shifts at 58.2 ppm (Sb) and 82.2 ppm (Bi), which are downfield from known main-group element Pn–PCO compounds (−441 to −225.8 ppm).^3a^ Two doublets were observed in the ^13^C NMR spectra for both the antimony [203.1 ppm (^1^*J*_CP_ = 76.0 Hz) and 148.8 ppm (^2^*J*_CP_ = 52.8 Hz)] and bismuth [203.6 ppm (^2^*J*_CP_ = 81.7) and 152.0 ppm (^1^*J*_CP_ = 49.6 Hz)] complexes.

**Figure 3:**
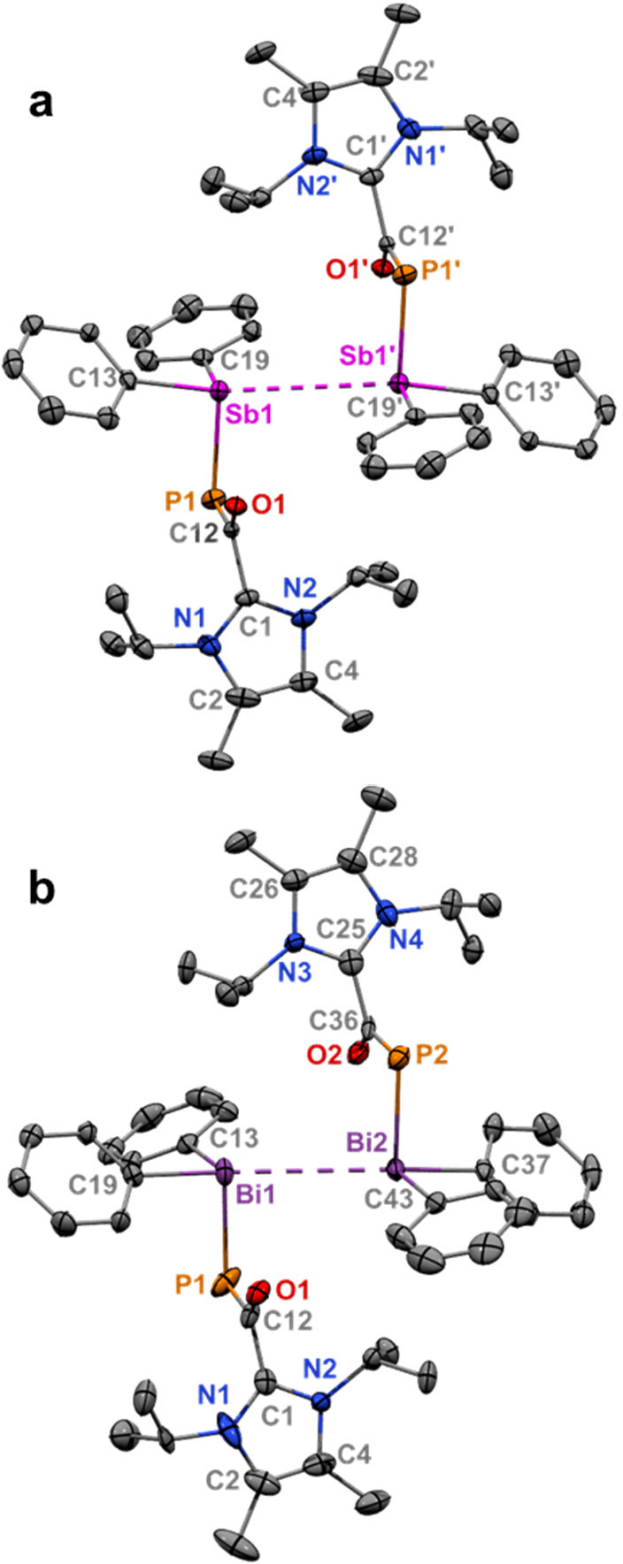
Molecular structure for **3** (a): Thermal ellipsoids at 50% probability; H atoms omitted for clarity. Selected bond distances (Å) and angles (deg): Sb1–C13 2.154(6); Sb1–C19 2.155(6); Sb1–P1 2.5042(16); Sb1–Sb1′ 3.9619(17); P1–C12 1.748(6); O1–C12 1.264(7); C1–C12 1.529(7). C13–Sb1–C19 97.7(2); C13–Sb1–P1 99.79(15); C19–Sb1–P1 91.77(15); Sb1′–Sb1–P1 94.602(40); Sb1′–Sb1–C19 97.237(158); Sb1′–Sb1–C13 158.899(168). Molecular structure for **4** (b): Thermal ellipsoids at 50% probability; H atoms were omitted for clarity. Selected bond distances (Å) and angles (deg): Bi1–C19 2.229(6); Bi1–C13 2.272(7); Bi1–P1 2.589(2); Bi1–Bi2 3.8204(6); Bi2–C37 2.251(7); Bi2–C43 2.257(8); Bi2–P2 2.594(2); P1–C12 1.736(7); P2–C36 1.746(8); O1–C12 1.255(8); O2–C36 1.258(8); C1–C12 1.525(11); C25–C36 1.515(11). C19–Bi1–C13 94.2(2); C19–Bi1–P1 97.38(19); C13–Bi1–P1 87.26(19); C37–Bi2–C43 94.8(3); C37–Bi2–P2 98.56(19); C43–Bi2–P2 90.99(19); Bi2–Bi1–P1 86.913(49); Bi2–Bi1–C13 101.940(176); Bi2–Bi1–C19 163.528(178); Bi1–Bi2–P2 89.365(43); Bi1–Bi2–C43 104.176(179); Bi1–Bi2–C37 159.346(177).

Single crystals of compounds **3** and **4** were obtained by layering the original THF filtrate with hexanes in a 1:1 ratio at −37 °C. Interestingly, the molecular structure revealed that the NHC transferred from the pnictogen center to the phosphaketene (Figure 3). Compounds **3** and **4** are unstable at room temperature and −37 °C, respectively, and decompose slowly in the solid state after a few days. The formation of these products is consistent with the ^13^C NMR spectra. A stretching frequency was not observed for the carbonyl group in the IR spectrum; however, this is consistent with reported NHC–phosphaketenyl species.^4d^ The solid-state structures of **3** and **4** reveal pnictogen centers in a seesaw environment with intermolecular Pn–Pn interactions at 3.9619(17) Å and 3.8204(6) Å, respectively. The Pn–P bond lengths for both **3** (2.5042(16) Å) and **4** (2.589(2)–2.594(2) Å) are close to the sum of covalent radii for Sb and P (*R*_SbP_ = 2.50 Å), as well as for Bi and P (*R*_BiP_ = 2.61 Å).^19^

**Scheme 3:**
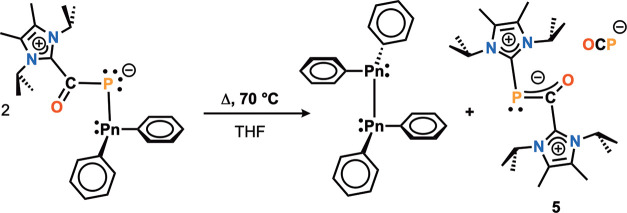
Thermal Reduction at Sb or Bi Center to Tetraphenyldipnictines and [(NHC)_2_OCP][OCP]

Recently, Grützmacher and co-workers demonstrated that *N*-heterocyclic carbene (NHC)–phosphaketene adducts of Ph_3_Sn—P=C=O and Ph_3_Ge—P=C=O undergo a decarbonylation reaction when heated to form the phosphenidinyl complexes NHC–P–SnPh_3_ and NHC–P–GePh_3_.^4d^ We were therefore interested in probing the thermal reactivity of compounds **3** and **4** (Scheme 3), which can be considered group 15 analogues of the aforementioned Sn and Ge phosphaketene complexes. Compound **3** was heated to 90 °C for 24 h in a J-Young NMR tube. The peaks in this ^1^H NMR spectrum matched those reported in the literature for tetraphenyldistibine (Figure S14).^16s^ Compound **4**, being less stable than **3**, was heated at 70 °C for 3 h in C_6_D_6_. Free NHC emerged along with 100% conversion to tetraphenyldibismuthine (Figure S15). Single crystals suitable for X-ray diffraction were grown from C_6_D_6_ inside the NMR tube. The solid-state structure revealed a new polymorph of tetraphenyldibismuthine (**6**) (Figure S19). It is noteworthy that compounds **3** and **4** slowly covert to **5** and tetraphenyldipnictogen at room temperature; therefore, heat was applied to escalate the reactions as described.

In addition to the formation of tetraphenyldipnictine, an orange solid precipitated from the C_6_D_6_ solution. The orange solid is insoluble in most common organic solvents except for dichloromethane but decomposes within an hour after dissolution. The ^1^H NMR spectrum of the orange solid revealed one broad and one well-defined heptet, suggesting two distinct NHC ligand environments. The ^31^P NMR showed a broad singlet at 22.1 ppm and a sharp singlet at −395.1 ppm. The latter shift closely resembles the resonance of 2-phosphaethynolate in D_2_O (−396.4 ppm).^3b^ Four doublets (δ = 200.7, 170.2, 150.2, 146.0) were observed in the ^13^C NMR spectrum. Further supporting our assignment, the signal at 170.2 ppm (^1^*J*_CP_ = 63.4 Hz) agrees well with known ^13^C NMR shifts for 2-phosphaethynolate, while the other signals are attributed to three new ^13^C–^31^P coupling environments. Similar to compounds **3** and **4**, no signals were observed in the IR spectrum for the CO stretch in the cationic unit of **5**. Two different stretches were observed for the phosphaalkyne at 1788 and 1768 cm^–1^, resulting from different orientations of [OCP]^−^ in the solid-state structure.

**Figure 4:**
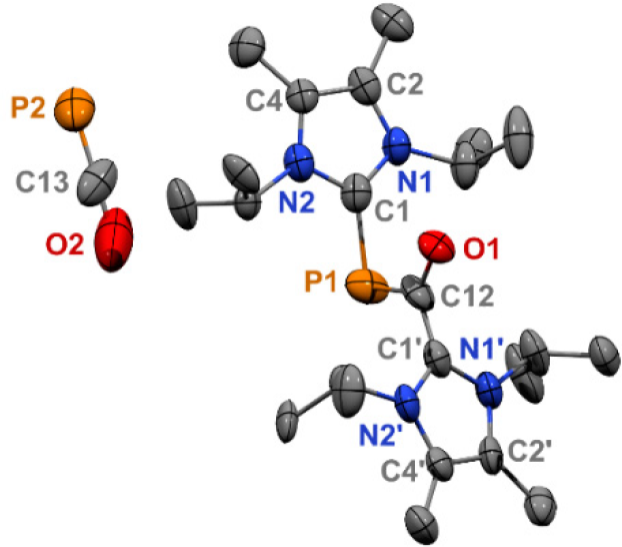
Molecular structure of **5**: Thermal ellipsoids at 50% probability; H atoms omitted for clarity. Only one orientation of the symmetry disordered [OCP]^−^ anion is shown. Selected bond distances (Å) and angles (deg): C1–P1 1.890(6); P1–C12 1.755(14); C12–O1 1.268(12); C12–C1′ 1.421(16). C1–P1–C12 98.3(5); O1–C12–C1′ 117.0(14).

Orange single crystals of compound **5** suitable for X-ray diffraction were obtained by heating a THF solution of **3** at 55 °C overnight. The crystal structure shows a cation containing two NHCs coordinated to a [OCP] core with an [OCP]^−^ counteranion (Figure 4). A 2-fold rotation axis perpendicular to the P1–C12 bond in the cation causes the two halves of the molecule to be disordered by symmetry in the solid state. This symmetry results in identical bond lengths and angles for both NHC ligands. A similar disorder exists in the anion. There are currently eight other molecular structures containing uncoordinated [OCP]^−^ counter-anions reported in the CSD database.^4c,6c,6d,20^ The C1–P1 bond (1.890(6) Å) is longer than those in neutral NHC_2_P_2_ complexes (1.750–1.754 Å)^21^ and cationic [NHC_2_P_2_]^+^ complexes (1.795–1.841 Å).^17c,21a,22^

To gain insights into the formation mechanism leading to the new compounds and the bonding situation thereof, we carried out DFT calculations employing the ωB97XD range separated functional with the def2-SVP and def2-TZVP basis sets, which is similar to the level of theory used previously to describe the bonding in carbene complexes of bismuth.^17b^ Relevant energies and structural parameters are shown in Table 1.

**Table 1:** Complex Formation Energies (Δ*E*) and Gibbs Free Energies (Δ*G*)^a^, Geometrical Parameters^b^, NPA Partial Charges of Pn (*q*) in Electrons, and Net Charge Transfer in Electrons (Δ*q*) at the ωB97XD/def2-TZVP Level

compound	**1**	**2**	NHC–SbPh_2_PCO	NHC–BiPh_2_PCO	**3**	**4**
Δ*E*	–23.9	–26.2	–19.2	–21.5	–29.7	–29.2
Δ*G*	–9.4	–11.7	–3.0	–7.1	–12.4	–12.0
d(Pn–C_carbene_/C–C_carbene_)	2.572	2.715	2.638	2.801	1.513	1.513
WBI(Pn–C_carbene_/C–C_carbene_)	0.37	0.31	0.32	0.26	0.93	0.93
*q*(Pn)	1.182	1.246	1.058	1.121	0.899	0.936
Δ*q*	0.235	0.202	0.226	0.186	0.755	0.751

a In kcal/mol.

b Bond length in Å/Wiberg bond indicies.

**Figure 5:**
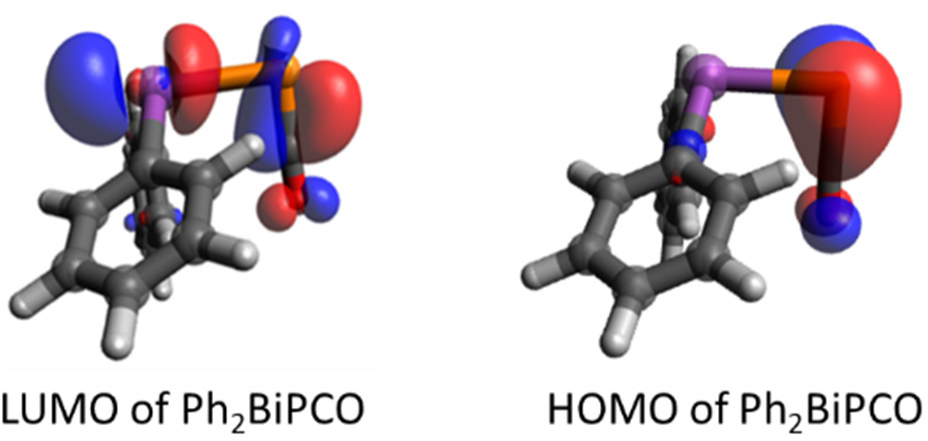
LUMO and HOMO of Ph_2_BiPCO.

**Figure 6:**
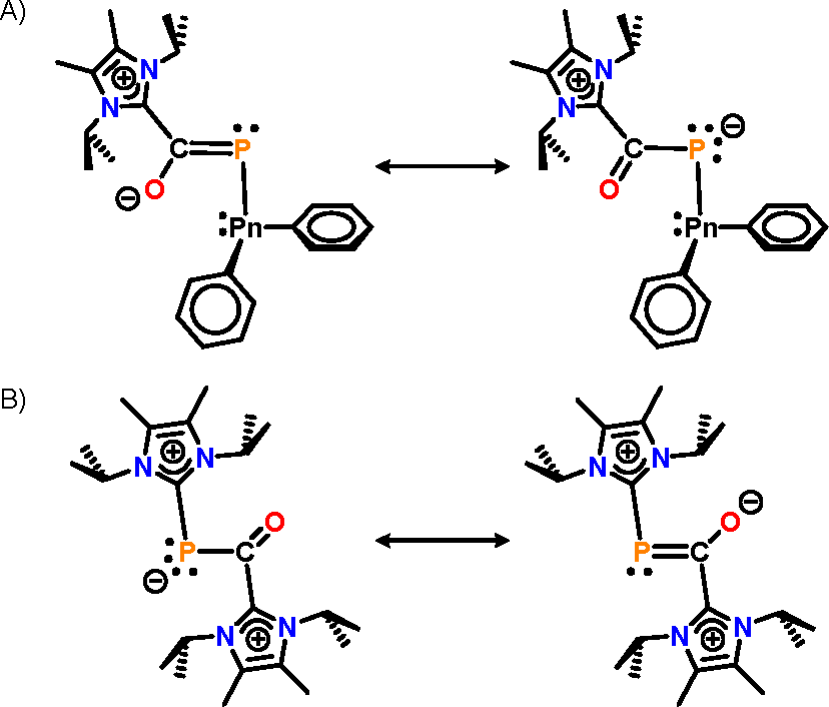
Resonance structures for compounds **3**/**4** (A) and cation **5** (B).

**Figure 7:**
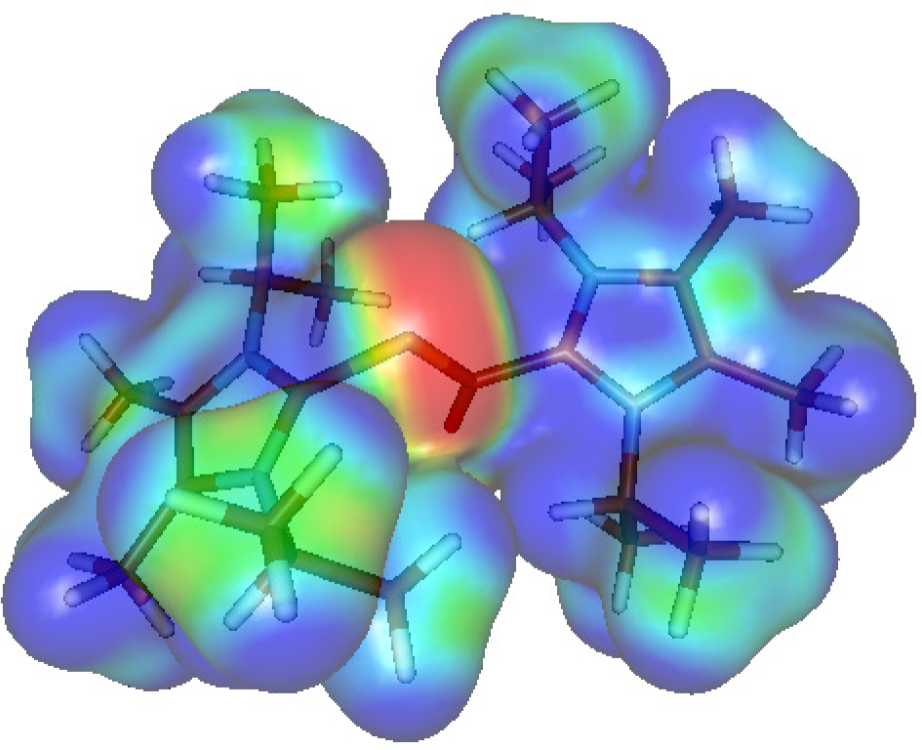
Molecular electrostatic potential for the cationic moiety of **5**.

The complex formation energy leading to adduct **2** is −26.2 kcal/mol (calculated with respect to the isolated carbene and diphenyl bismuth chloride). This value is greater than the values of −35.9 to −44.6 kcal/mol reported for NHC and CAAC complexes of PhBiCl_2_,^18b^ a stronger Lewis acid owing to the presence of two chlorine atoms instead of one in Ph_2_BiCl. This agrees nicely with the observations above on the solid-state structures, which revealed rather long ^NHC^C–Pn bonds as a result of a weaker interaction. Compared to **2**, the antimony analogue **1** is slightly less stable (Δ*E* = −23.9 kcal/mol), explainable by the weaker electron pair accepting property of antimony than that of bismuth. The same phenomenon is observed for the NHC–Ph_2_PnPCO complexes, which are assumed as possible intermediates during the replacement of the chlorides of **1** and **2** by phosphaethynolate anion. However, the phosphaketene complexes are destabilized compared to their chloro-analogues, due to the lower electronegativity of P compared to Cl. Indeed, the partial charge at the Bi center in the uncomplexed Ph_2_BiPCO and Ph_2_BiCl is +1.010 and +1.226 e, respectively, in line with the lower Lewis acidity of the former compared to the latter. The reduced stability of the phosphaketene complexes compared to analogous chloro-complexes is accompanied by the weakening of the ^NHC^C–Pn bonds; these bonds are longer and their Wiberg bond indices (WBI), accounting for the covalent character, are lower. Thus, the net charge transfer is smaller. The LUMO of Ph_2_BiPCO (Figure 5) shows main contributions both at the Bi center and the carbon atom of the PCO moiety, explaining why this species may be complexed either at Bi or on the phosphaketenyl carbon center. The rearranged phosphaketene carbene adducts **3** and **4**, in which the carbene is coordinated to the PCO carbon atom, are significantly more stable than the Pn-coordinated analogues. Thus, the driving force for the carbene migration is the formation of a stronger C–C bond instead of a dative C–Pn bond. These C–C bonds show a high covalent character (WBI: 0.93) and remarkable net charge transfer from the carbene to the PCO moiety of Δ*q* = 0.751 and 0.755, meaning that the carbenic unit possesses a large partial positive charge. Furthermore, the WBIs of PC/CO bonds (1.41/1.48 and 1.42/1.47 for **3** and **4**, respectively) indicate delocalization in the PCO fragment. Hence, the structure of the C-coordinated Ph_2_PnPCO adducts **3** and **4** can be best described as a superposition of two zwitterionic resonance structures (Figure 6A). We also studied the electronic structure and bonding of the cationic fragment of compound **5**. Even though [(NHC)_2_OCP]^+^ can be regarded formally as an adduct of a cationic OCP^+^ unit and two carbenes, the NPA charges and WBI values suggest the positive charge is localized on the NHC ligands (Figure 6B). While the sum of charges in the OCP core is −0.375e, both NHC fragments possess high partial charges of 0.804e and 0.571e. The WBI of the P–C(carbene) and C–C(carbene) bonds of 0.93 indicate covalent character, and the PC/CO bonds show a delocalization in the OCP moiety. The bis-zwitterionic charge distribution of the [(NHC)_2_OCP]^+^ cation is also visible on the molecular electrostatic potential (Figure 7).

We also aimed to understand the formation of the Ph_2_PnPnPh_2_ dimers and compound **5**; therefore, we investigated possible reaction mechanisms by means of computations. As the reactivity of **3** and **4** are rather similar, we focused on the bismuth analogue. Because this reaction proceeds in C_6_D_6_, the gas phase approximation seems to be appropriate without solvent effects. In the following, we discuss the energies obtained at the ωB97XD/def2-SVP level.

**Figure 8:**
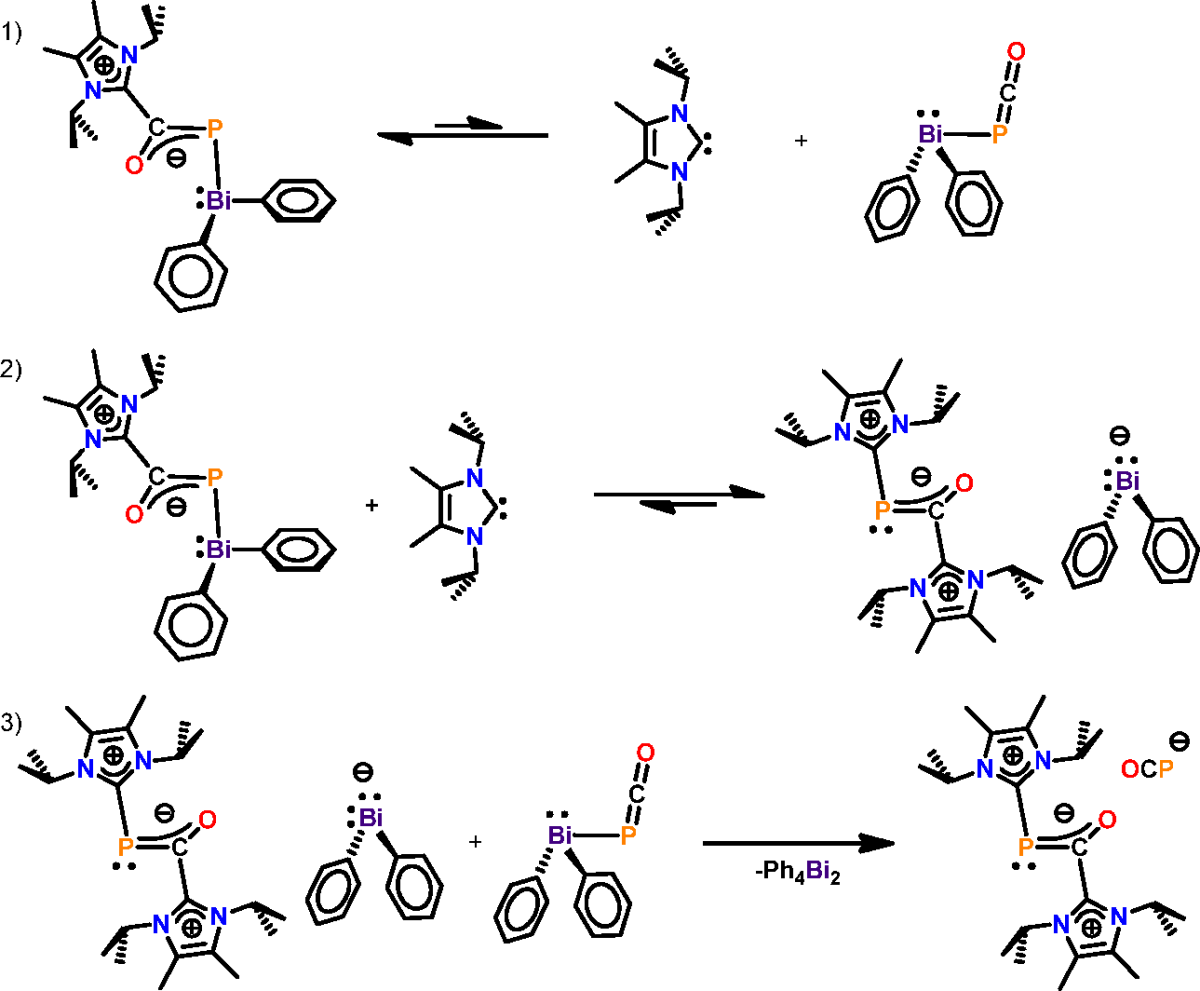
Proposed mechanism for the formation of [(NHC)_2_OCP]^+^[OCP]^−^.

**Figure 9:**
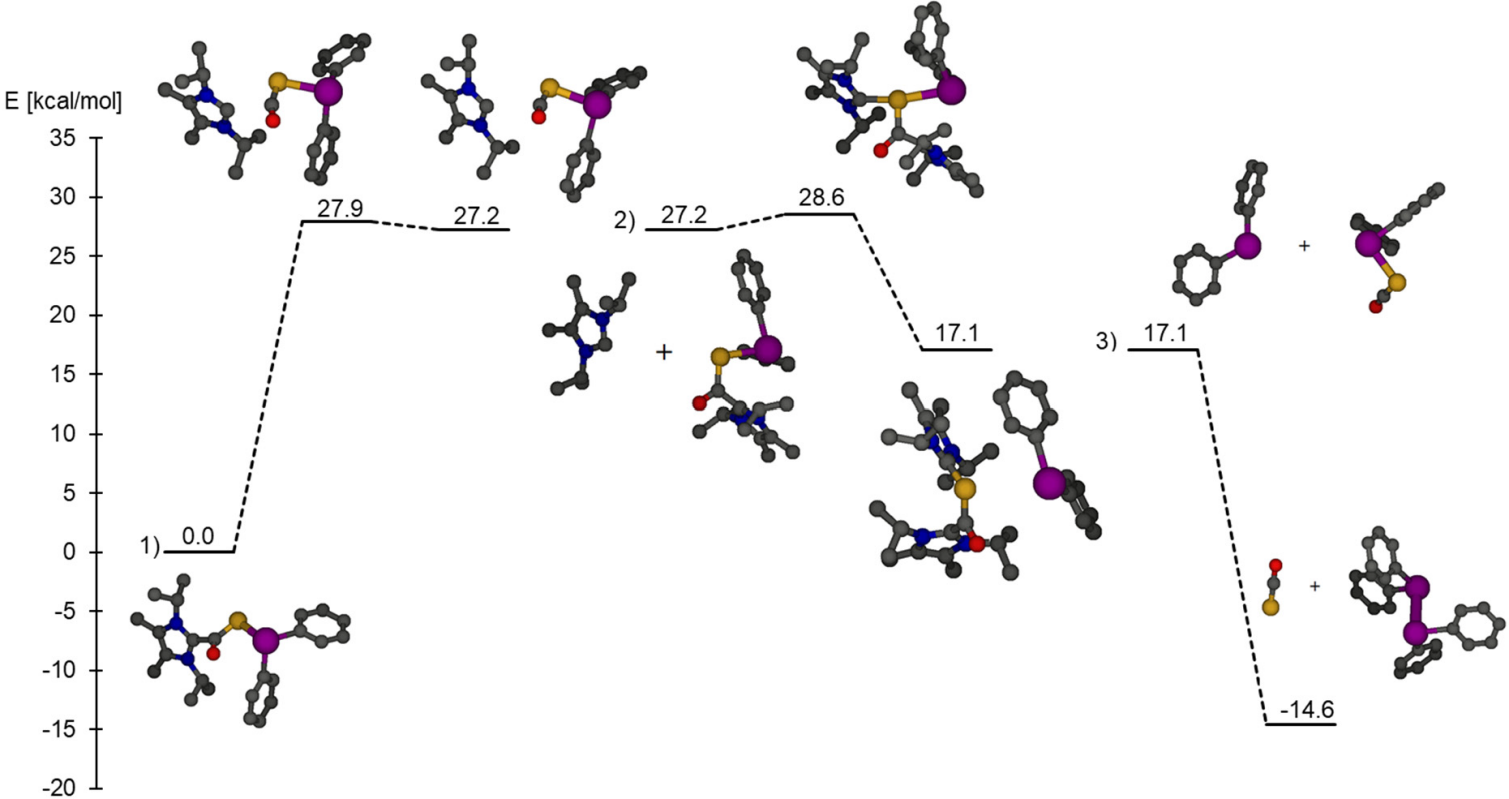
Energy profile for the decomposition of **4** leading to **5**. The three mechanistic steps illustrated in Figure 8 proceed on different potential energy surfaces. For easier understanding, the energy levels of each initial mechanistic step are shifted to the energy of the previous step. (Atom colors: C, black; O, red; N, blue; P, orange; Bi, purple). In step 3 the countercation is not shown for clarity but was included in the computations.

The formation of the tetraphenyldibismuthine may indicate a radical mechanism, in which the first step would be the homolytic dissociation at the P–Bi bond of adduct **4**, or alternatively, the free Ph_2_BiPCO. However, both reactions are highly endothermic (*ΔE* = 53.0 and 51.7 kcal/mol, respectively); thus, they are unlikely to happen even at higher temperature. We considered further alternative pathways and a plausible mechanism (Figure 8). The first step of the reaction is the partial dissociation of adduct **4**, resulting in the free carbene and Ph_2_BiPCO. This reaction is rather endothermic and proceeds via an activation barrier of 27.9 kcal/mol (Figure 9), resulting in a weakly bound complex of NHC and Ph_2_BiPCO at the energy of 27.2 kcal/mol. Even though this reaction is likely shifted toward the side of the starting adduct, the formation of small amounts of free carbene is expected, especially if the entropy factor is taken into account (dissociation Gibbs free energy: 18.9 kcal/mol). This is further supported by the experimental observation of uncoordinated NHC during the reaction. The second step of the reaction is an attack of the free carbene onto the P center of adduct **4**, delivering the contact ion pair of the [(NHC)_2_OCP]^+^ cation with a diphenyl bismuthide ([BiPh_2_]^−^) counteranion. The nucleophilic substitution at the phosphaketene P center is known in the literature, and it has been shown that the attack of Lewis bases (L) on the phosphorus center of phosphanyl phosphaketenes R—P=C=O results in the adduct R—P=L and carbon monoxide. In our case, however, the C of the PCO unit is occupied by the carbene fragments; thus, the decarbonylation is hampered. Instead, the bismuthide anion is released in a slightly exothermic reaction (Δ*E* = −10.1 kcal/mol). Since all of our attempts to locate the transition state of step 2 failed, we performed a relaxed optimization scan connecting the structures at the two sides of the equation and estimated a barrier of 1.4 kcal/mol via this approach. The thermodynamic sink is obtained in reaction step 3, which is strongly exothermic with a reaction energy of Δ*E* = −31.7 kcal/mol. Since we could not locate any transition states for this step, we performed a relaxed scan computation which showed a continuous decrease in the energy; therefore, this reaction step is assumed to proceed without barrier. In this final step, the attack of the [BiPh_2_]^−^ anion at the Bi center of Ph_2_BiPCO formed in step 1 delivers the dibismuthine Ph_2_BiBiPh_2_ as well as the [OCP]^−^ anion for compound **5**.

## Conclusion

The reaction of Ph_2_PnCl with Na[OCP] results in the formation of tetraphenyldipnictine and an insoluble unidentifiable product. Therefore, we prepared the respective NHC-supported Ph_2_PnCl compounds (**1** and **2**) and explored their reactivity with Na[OCP]. In both cases, the NHC transfers from the pnictogen center to the phosphaketene carbon atom. The crystal structures of these two OCP complexes reveal significant metal–metal interactions. Heating the NHC–phosphaketene adducts **3** and **4** results in a formal reduction at the pnictogen center, Pn^III^ to Pn^II^, resulting in the formation of tetraphenyldipnictine and [(NHC)_2_OCP]^+^[OCP]^−^ (**5**). Notably, compound **5** represents the first example of an ionic compound where the cation and anion each possess an OCP unit. These results further demonstrate the utility of the 2-phosphaethynolate ion as a reductant and contrast with the chemistry observed for the group 14 (Sn and Ge) analogues, which undergo decarbonylation to yield phosphinidenyl species.

## Experimental Section

### General Considerations

All reactions were carried out under an atmosphere of purified argon in a MBRAUN LABmaster glovebox equipped with a −37 °C freezer. All solvents were distilled over sodium/benzophenone. Glassware was oven-dried at 190 °C overnight. Deuterated solvents were purchased from Acros Organics and Cambridge Isotope Laboratories and were dried the same way as their protic analogues. The NMR spectra were recorded at room temperature on a Varian Inova 500 MHz (^1^H: 500.13 MHz and ^31^P: 202.46 MHz) and a Bruker Avance 800 MHz spectrometer (^1^H: 800.13 MHz, ^13^C: 201.19 MHz). ^1^H and ^13^C chemical shifts are reported in parts per million (ppm) and are referenced using the residual proton and carbon signals of the deuterated solvent (^1^H: C_6_D_6_, δ 7.16; ^13^C: C_6_D_6_, δ 128.06; ^1^H: THF-*d*_8_, δ 3.58, 1.72; ^13^C: THF-*d*_8_, δ 67.21, 25.31). ^31^P NMR chemical shifts are reported in ppm and are referenced externally to an 85% H_3_PO_4_ solution. Elemental analyses were performed at the University of Virginia and Midwest Microlab, 7212 North Shadeland Avenue, Suite 110, Indianapolis, IN 46250, USA. Single-crystal X-ray diffraction data were collected on a Bruker Kappa APEXII Duo system. An Incoatec Microfocus IμS (Cu Kα, λ = 1.54178 Å) and a multilayer mirror monochromator were used for **1**, **3**, and **4**, and a fine-focus sealed tube (Mo Kα, λ = 0.71073 Å) and a graphite monochromator were used for **2**, **5**, and **6**. The frames were integrated with the Bruker SAINT software package^21^ using a narrow-frame algorithm. Data were corrected for absorption effects using the Multi-Scan method.^21^ The structures were solved and refined using the Bruker SHELXTL software package^22^ within APEX3^21^ and OLEX2.^23^ Non-hydrogen atoms were refined anisotropically. Hydrogen atoms were placed in geometrically calculated positions with *U*_iso_ = 1.2*U*_equiv_ of the parent atom (*U*_iso_ = 1.5*U*_equiv_ for methyl). For **3**, CELL_NOW^24^ was used to identify a two-component twin. Starting with 1058 reflections, 889 reflections were fit to the first domain and 441 to the second domain (165 exclusively), with 4 unindexed reflections remaining. The twin domain was oriented at a 179.9° rotation about the reciprocal axis 0.003 0.500 1.000. The twin law was −0.994 0.009 0.004/0.622 −0.318 0.657/1.257 1.364 0.311. The structure was refined as a two-component twin on HKLF5 data, with the BASF for the twin domain refining to 0.12525. One isopropyl group was found to be disordered over two positions. The relative occupancy was freely refined, and constraints were used on the anisotropic displacement parameters of one pair of disordered atoms. For **4**, the relative occupancies of the disordered isopropyl groups were freely refined. Constraints were used on the anisotropic displacement parameters of the disordered C6/C6a pair. For **5**, one isopropyl group was disordered over two positions. The relative occupancy was freely refined and restraints were used on the anisotropic displacement parameters of the disordered atoms. The OCP unit connecting the two carbenes was disordered by symmetry and was therefore modeled at 50% occupancy. The outer-sphere [OCP]^−^ anion was disordered over two positions, each of which was located on a symmetry element. The relative occupancies of the different orientations were freely refined, at 50% occupancy to account for the symmetry and then with restraints on the anisotropic displacement parameters and bond lengths of the disordered atoms.

### Synthesis of (NHC)SbPh_2_Cl (**1**)

To a 20 mL scintillation vial, Ph_2_SbCl (690 mg, 2.22 mmol) was added and stirred in toluene (5 mL). A toluene solution (5 mL) of NHC (400 mg, 2.22 mmol) was added, and then, the reaction was allowed to stir for 1 h. After the filtration, the crude solid was washed with hexanes and then dried *in vacuo*. Compound **1** was obtained as a white solid (925 mg, 85%). Crystals suitable for X-ray diffraction studies were obtained from a toluene/hexane mixture at −37 °C. ^1^H NMR (C_6_D_6_, 500.13 MHz): δ 8.17 (t, 4H, C*H*_*ortho*_), 7.22 (t, 4H, C*H*_*meta*_), 7.12 (t, 2H, C*H*_*para*_), 4.69 (Br, 2H, C*H*(CH_3_)_2_), 1.56 (s, 6H, C(backbone)–C*H*_3_), 0.81 (s, 12H, CH(C*H*_3_)_2_). ^13^C{^1^H} NMR (THF-*d*_8_, 201.193 MHz): δ 146.53 (C_Ph-*i*_), 136.50 (C_Ph-*o*_), 128.51 (C_Ph-*m*_), 128.23 (C_Ph-*p*_), 125.33 (C_vinyl_), 52.34 (N–*C*H–(CH_3_)_2_), 21.23 (N–CH–(*C*H_3_)_2_), 9.74 (C_vinyl_–*C*H_3_). Anal. calcd for C_23_H_30_N_2_SbCl: C, 56.18; H, 6.15; N, 5.70%. Found: C, 55.95; H, 6.22; N, 5.68%.

### Synthesis of (NHC)BiPh_2_Cl (**2**)

To a 20 mL scintillation vial, Ph_2_BiCl (1.111 g, 2.77 mmol) was added and stirred in toluene (5 mL). A toluene solution (5 mL) of NHC (500 mg, 2.77 mmol) was added, and then, the reaction was allowed to stir for 1 h. After the filtration, the crude solid was washed with hexanes and then dried *in vacuo*. Compound **2** was obtained as a white solid (1.51 g, 94%). Colorless crystals suitable for X-ray diffraction studies were obtained from a toluene/hexane mixture at −37 °C. ^1^H NMR (THF-*d*_8_, 500.13 MHz): δ 8.35 (br, 4H, C*H*_*ortho*_), 7.41 (t, *J* = 7.6 Hz, 4H, C*H*_*meta*_), 7.21 (t, *J* = 7.3 Hz, 2H, C*H*_*para*_), 4.51 (hept, *J* = 6.7 Hz, 2H, C*H*(CH_3_)_2_), 2.15 (s, 6H, C(backbone)–C*H*_3_), 1.17 (d, *J* = 7 Hz, 12H, CH(C*H*_3_)_2_). ^13^C{^1^H} NMR (THF-*d*_8_, 201.19 MHz): δ 139.35 (C_Ph-*o*_), 131.45 (C_Ph-*m*_), 128.08 (C_Ph-*p*_), 126.48 (C_vinyl_), 54.12 (N–*C*H–(CH_3_)_2_), 22.75 (N–CH–(*C*H_3_)_2_), 10.28 (C_vinyl_–*C*H_3_). Anal. calcd for C_23_H_30_N_2_BiCl: C, 47.72; H, 5.22; N, 4.84%. Found: C, 47.37; H, 5.41; N, 4.77%.

### Synthesis of NHC–C(O)P-SbPh_2_ (**3**)

To a 20 mL vial, (NHC)BiPh_2_Cl (97 mg, 0.197 mmol) was added and stirred in THF. Na[OCP]•(dioxane)_*x*_ (65 mg, 0.217 mmol) was added to the stirring solution. upon addition, the solution immediately turned yellow. After stirring for 5 min at room temperature, insoluble NaCl was removed by filtration, and the yellow THF solution was layered with hexanes in a 1:1 ratio and allowed to sit for 1 day at −37 °C. After removal of the solvent and drying in vacuo, the product was obtained as a yellow crystalline solid (50 mg, 49% yield). Note: compound **3** decomposes to **5** and Ph_4_Bi_2_ at room temperature. Note: compound **3** decomposes to **5** and Ph_4_Bi_2_ at −37 °C. ^1^H NMR (C_6_D_6_, 500.13 MHz) δ 8.18 (d, *J* = 7.9 Hz, 4H, C*H*_*ortho*_), 7.21–7.15 (m, 4H, C*H*_*meta*_), 7.12 (t, *J* = 7.3 Hz, 2H C*H*_*para*_), 5.10 (hept, *J* = 6.9 Hz, 2H, C*H*(CH_3_)_2_), 1.34 (s, 6H, C(backbone)–C*H*_3_), 1.06 (d, *J* = 7.1 Hz, 12H, CH(C*H*_3_)_2_). ^13^C{^1^H} NMR (201.19 MHz, C_6_D_6_) δ 203.13 (d, *J* = 76.0 Hz, *C*=O), 148.84 (d, *J* = 52.8 Hz, C_NHC_), 141.01 (C_Ph-*i*_), 137.73 (C_Ph-*o*_), 128.28 (C_Ph-*m*_), 127.28 (C_Ph-*p*_), 123.30 (C_vinyl_), 51.16 (N-*C*H-(CH_3_)_2_), 21.08 (N–CH-(*C*H_3_)_2_), 9.25 (C_vinyl_-*C*H_3_). ^31^P{^1^H} NMR (202.46 MHz, C_6_D_6_) δ 58.18 (s, 1P). IR: ν = 3040, 2971, 2934, 2854, 1625, 1574, 1427, 1371, 1310, 1217, 1105, 1051, 928, 729, 697 cm^–1^. Suitable elemental analysis could not be obtained due to solid-state instability. Thus, purity was assessed by immediately collecting the ^1^H, ^13^C, and ^31^P NMR data of a freshly made sample of **3**.

### Synthesis of NHC–C(O)P-BiPh_2_ (**4**)

To a 20 mL vial, (NHC)BiPh_2_Cl (200 mg, 0.344 mmol) was added and stirred in THF. Na[OCP]·(dioxane)_*x*_ (302 mg, 0.344 mmol) was added to the stirring solution. Immediately upon addition, the solution turned yellow. After it stirred for 5 min at room temperature, insoluble NaCl was removed by filtration and the yellow THF solution was layered with hexanes in a 1:1 ratio and allowed to sit for 1 day at −37 °C. After removal of the solvent and drying *in vacuo*, the product was obtained as a yellow crystalline solid (92 mg, 48% yield). Note: Compound **4** decomposes to **5** and Ph_4_Bi_2_ at −37 °C. ^1^H NMR (C_6_D_6_, 500.13 MHz): δ 8.51 (d, *J* = 7.6 Hz, 4H, C*H*_*ortho*_), 7.25 (t, *J* = 7.5 Hz, 4H, C*H*_*meta*_), 7.20–7.13 (m, 2H, C*H*_*para*_), 5.13 (hept, *J* = 6.9 Hz, 2H, C*H*(CH_3_)_2_), 1.33 (s, 6H, C(backbone)–C*H*_3_), 1.06 (d, *J* = 7.1 Hz, 12H, CH(C*H*_3_)_2_). ^13^C{^1^H} NMR (201.19 MHz, C_6_D_6_): δ 203.58 (d, *J* = 81.7 Hz, *C*=O), 152.03 (d, *J* = 49.6 Hz, C_NHC_), 151.04 (C_Ph-*i*_), 140.05 (C_Ph-*o*_), 130.15 (C_Ph-*m*_), 126.75 (C_Ph-*p*_), 123.45 (C_vinyl_), 51.36 (N–*C*H–(CH_3_)_2_), 21.37 (N–CH–(*C*H_3_)_2_), 9.46 (C_vinyl_–*C*H_3_). ^31^P{^1^H} NMR (202.46 MHz, C_6_D_6_): δ 82.17 (s, 1P). IR: ν = 3033, 2975, 2932, 2869, 1625, 1569, 1418, 1371, 1312, 1217, 1110, 995, 928, 723, 697 cm^–1^. Suitable elemental analysis could not be obtained due to solid-state instability. Thus, purity was assessed by immediately collecting the ^1^H, ^13^C, and ^31^P NMR data of a freshly made sample of **4**.

### Synthesis of [NHC–PC(=O)–(NHC)][OCP] (**5**)

To a 20 mL scintillation vial, (NHC)BiPh_2_Cl (505 mg, 869 μmol) was added and suspended in 10 mL of dry THF. Na[OCP]·(dioxane)_*x*_ (262 mg, 869 μmol) was added to the suspension, and the suspension was shaken vigorously for 1 min. The reaction mixture was then extracted into a 100 mL Schlenk tube. Orange crystals of [(NHC)_2_OCP][OCP] formed from the solution after sitting undisturbed at 55 °C overnight (106 mg, 51%). ^1^H NMR (500.13 MHz, CD_2_Cl_2_): δ 5.35 (br, 4.99, 2H, C*H*(CH_3_)_2_) (hept, *J* = 7.0 Hz, 2H, C*H*(CH_3_)_2_), 2.38 (s, 6H, C(backbone)–C*H*_3_), 2.35 (s, 6H, C(backbone)–C*H*_3_), 1.62 (d, *J* = 7.1 Hz, 12H, CH(C*H*_3_)_2_), 1.58 (d, *J* = 7.1 Hz, 12H, CH(C*H*_3_)_2_). ^13^C{^1^H} NMR (201.19 MHz, CD_2_Cl_2_): δ 200.71 (d, *J* = 64.2 Hz, *C*=O), 170.20 (d, *J* = 63.0 Hz, O*C*P), 150.24 (d, *J* = 86.9 Hz, *C*_NHC_–P), 146.04 (d, *J* = 67.5 Hz *C*_NHC_–C=O), 128.8 (C_vinyl_), 126.5 (C_vinyl_), 53.9 (N–*C*H–(CH_3_)_2_), 52.5 (N–*C*H–(CH_3_)_2_), 21.9 (N–CH–(*C*H_3_)_2_), 21.8 (N–CH–(*C*H_3_)_2_), 11.0 (C_vinyl_–*C*H_3_), 10.7 (C_vinyl_–*C*H_3_). ^31^P{^1^H} NMR (242.94 MHz, CD_2_Cl_2_): δ 22.72 (br, 1P, [OC*P*]^+^), −395.09 (s, 1P, [OC*P*]^−^). IR: ν = 3044, 2973, 2934, 2873, 1788, 1768, 1634, 1574, 1429, 1371, 1312, 1217, 1051, 930, 729, 729, 699 cm^–1^. Anal. calcd for C_24_H_40_N_4_O_2_P_2_: C, 60.24; H, 8.43; N, 11.71%. Found: C, 60.09; H, 8.52; N, 11.70%.

### Computational Details

The computations were carried out with the Gaussian 09 suite of programs.^23^ The structures were optimized using the ωB97XD functional in combination with the def2-SVP and the def2-TZVP basis sets. At each of the optimized structures vibrational analysis was accomplished to check whether the stationary point located is a minimum or a saddle point of the potential energy hypersurface. We neglected the solvent effect because toluene was used as the solvent. For Wiberg Bond Indexes and NPA charges, the NBO program version 5.0 was employed.^24^ The plotting of the orbitals was carried out with the AVOGADRO program (www.avogadro.cc).

## Supporting Information Available

NMR spectral data; IR spectral data; crystal structure of compound **6**; crystallographic refinement details; and computational details (PDF)

## Accession Codes

CCDC 1962839–1962843 and 1976639 contain the supplementary crystallographic data for this paper. These data can be obtained free of charge via www.ccdc.cam.ac.uk/data_request/cif, or by emailing data_request@ccdc.cam.ac.uk, or by contacting The Cambridge Crystallographic Data Centre, 12 Union Road, Cambridge CB2 1EZ, UK; fax: +44 1223 336033.
