# Supplementary material for: Indirect Access to Carbene Adducts of Bismuth- and Antimony-Substituted Phosphaketene and Their Unusual Thermal Transformation to Dipnictines and [(NHC)2OCP][OCP]
Source: Inorg Chem. 2021 Mar 9;60(7):4733–43. doi: 10.1021/acs.inorgchem.0c03683 (PMC8277130; doi:10.1021/acs.inorgchem.0c03683)
Supplement: Supplementary file 1 — ic0c03683_si_001.pdf [file ic0c03683_si_001.pdf]

Indirect Access to Carbene Adducts of Bismuth and Antimony Substituted Phosphaketene and their Unusual Thermal Transformation to Dipnictines and [(NHC)$_2$OCP][OCP]

Jacob E. Walley,[a] Levi S. Warring,[a] Erik Kertész,[b] Guocang Wang,[a] Diane A. Dickie,[a] Zoltán Benkő,[b*] Robert J. Gilliard, Jr.[a*]

[a]Department of Chemistry, University of Virginia, 409 McCormick Rd, PO Box 400319, Charlottesville, USA

[b]Department of Inorganic and Analytical Chemistry, Budapest University of Technology and Economics, Szent Gellért tér 4, H-1111 Budapest, Hungary

*To whom correspondence should be addressed: zbenko@mail.bme.hu; rjg8s@virginia.edu

Supporting Information

Table of Contents

NMR Spectral Data S2

IR Spectral Data S9

Crystal structure of compound 6 .... S11

Crystallographic Refinement Details S12

Computational Details S13

References S34

NMR Spectral Data

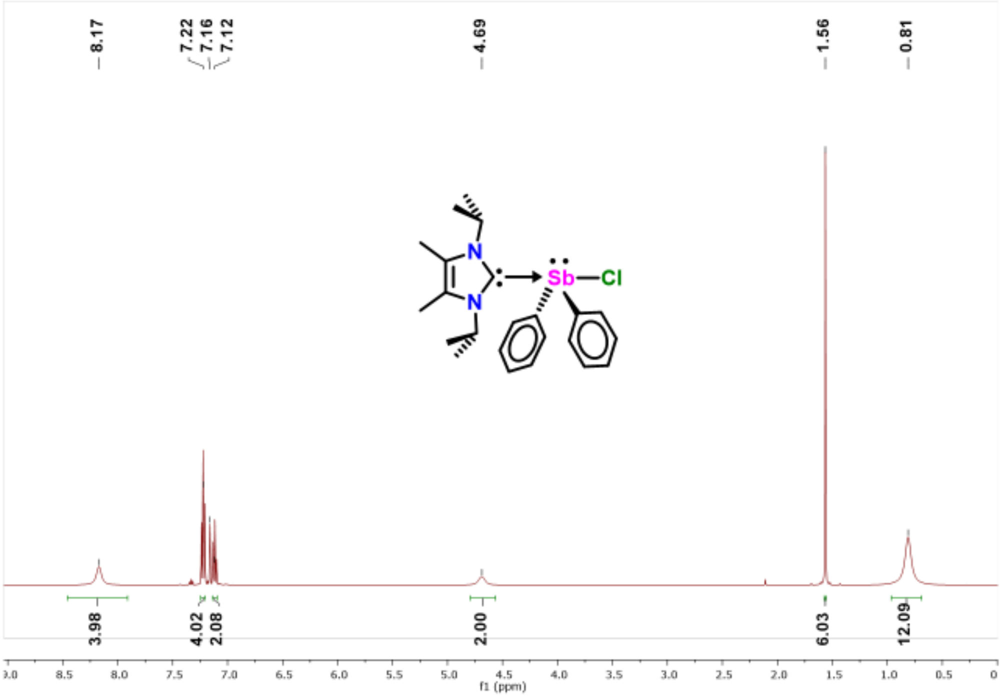

Figure S1: ^{1}H NMR spectrum (500.13 MHz, C_6D_6, 298 K) of complex **1**.

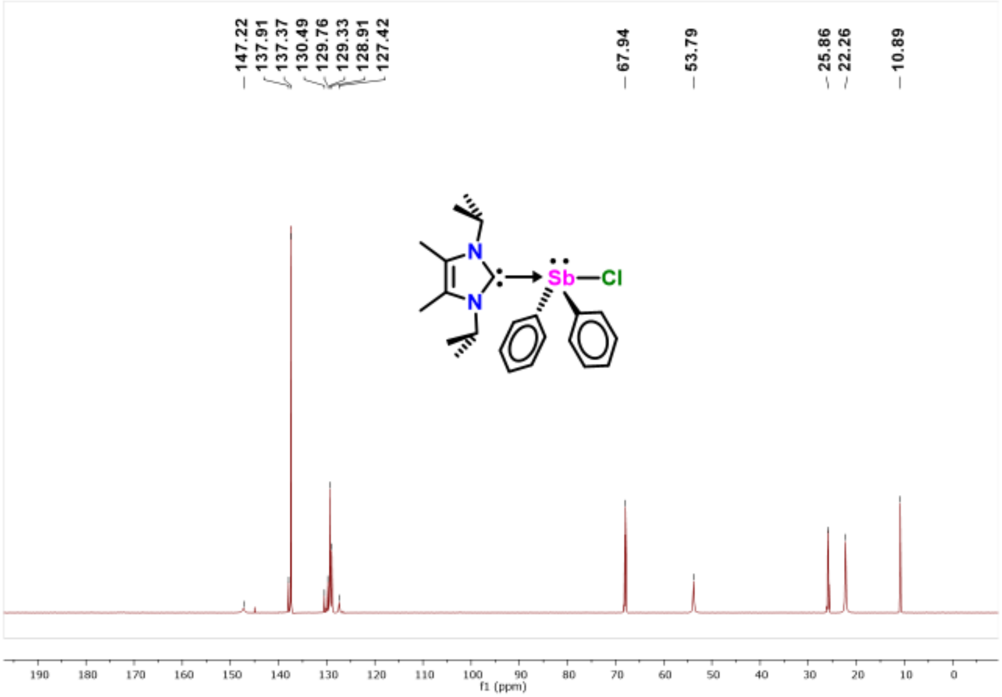

Figure S2: $^{13}C\{^{1}H\}$ NMR spectrum (201.19 MHz, C_6D_6, 298 K) of complex **1**.

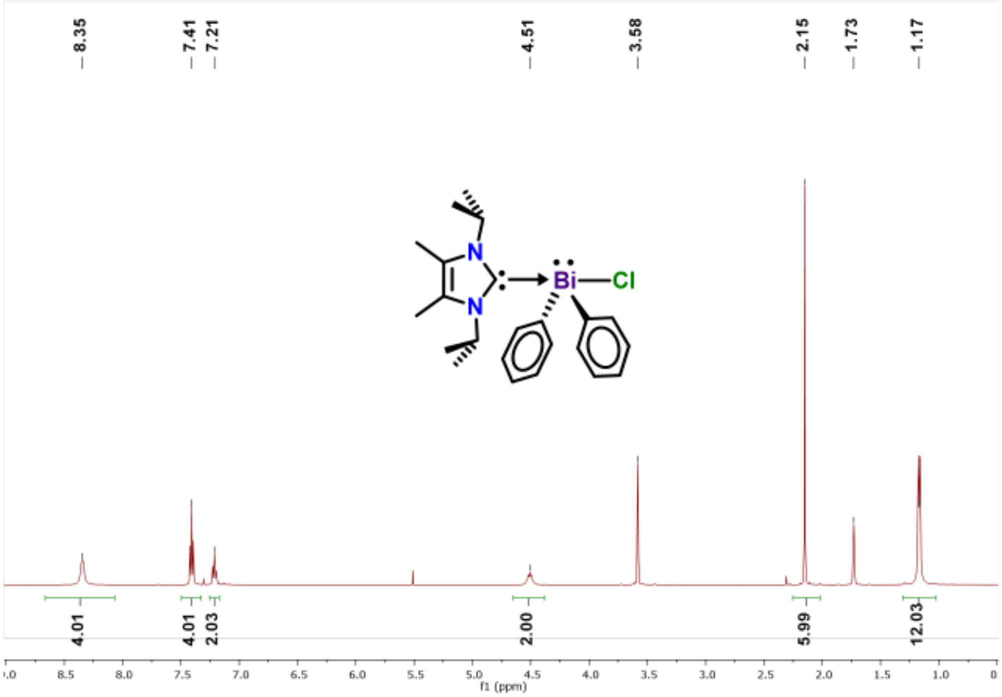

Figure S3: ^{1}H NMR spectrum (500.13 MHz, C_6D_6, 298 K) of complex **2**.

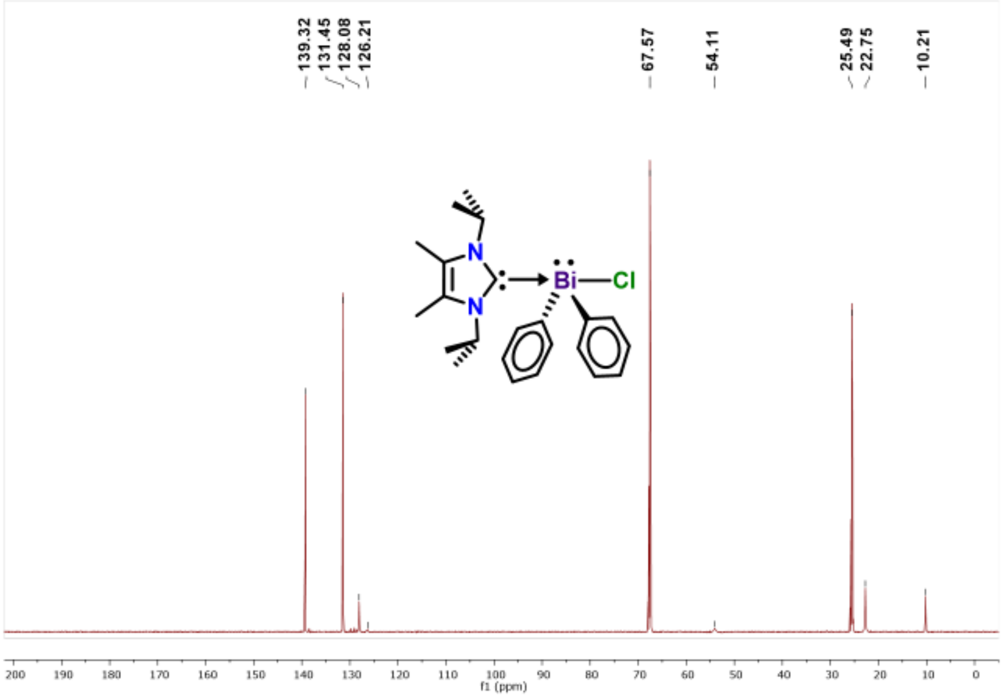

Figure S4: ^{13}C{^{1}H} NMR spectrum (201.19 MHz, C_6D_6, 298 K) of complex **2**.

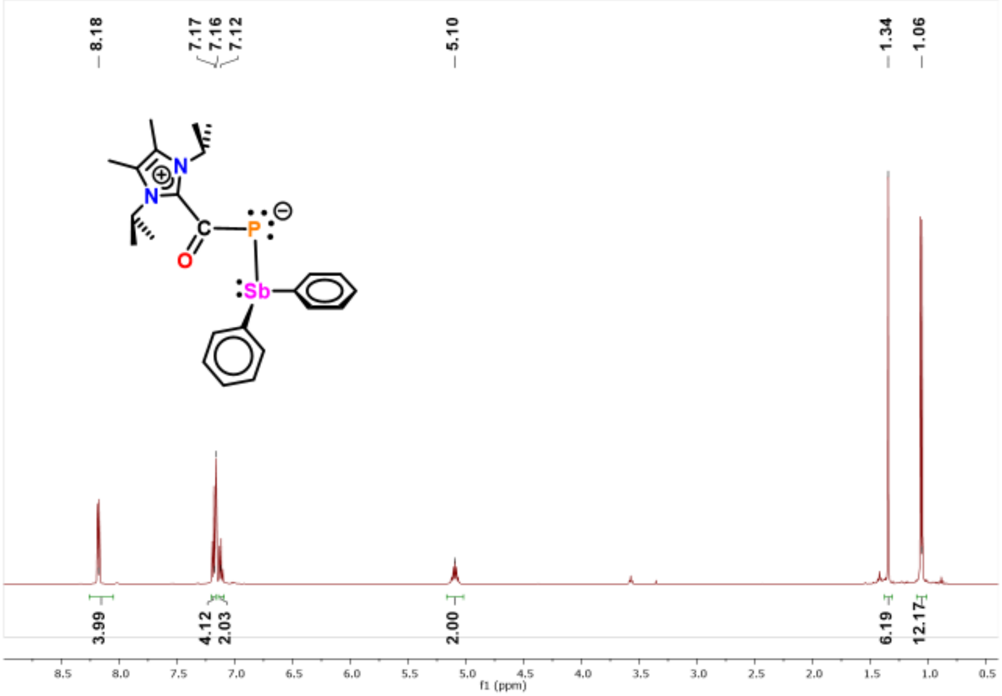

Figure S5: ^{1}H NMR spectrum (500.13 MHz, C_6D_6, 298 K) of complex **3**.

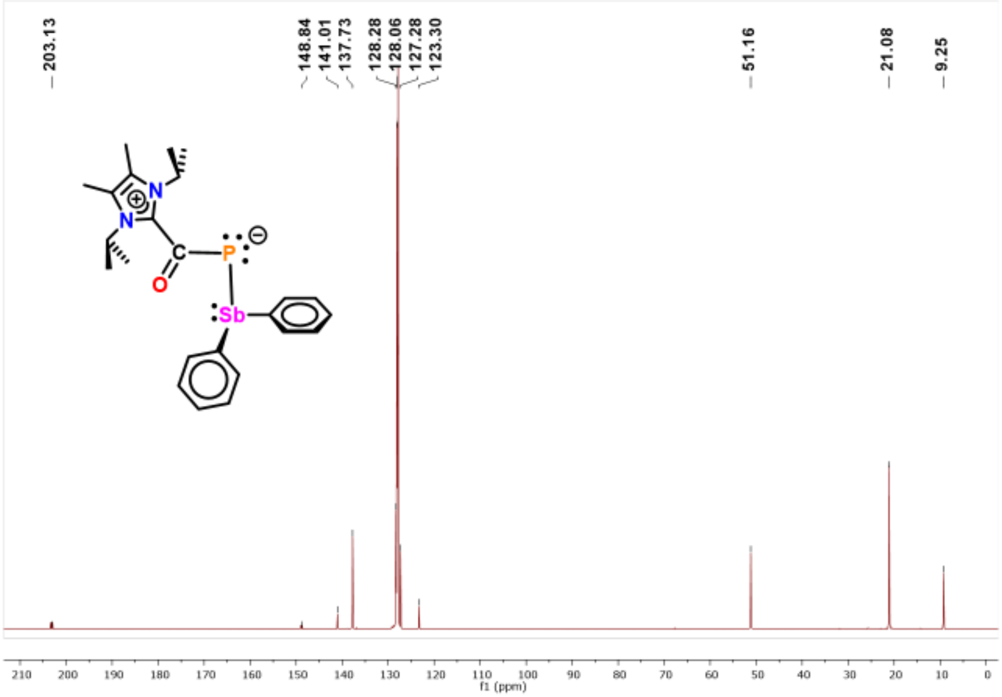

Figure S6: $^{13}C\{^{1}H\}$ NMR spectrum (201.193 MHz, C_6D_6, 298 K) of complex **3**.

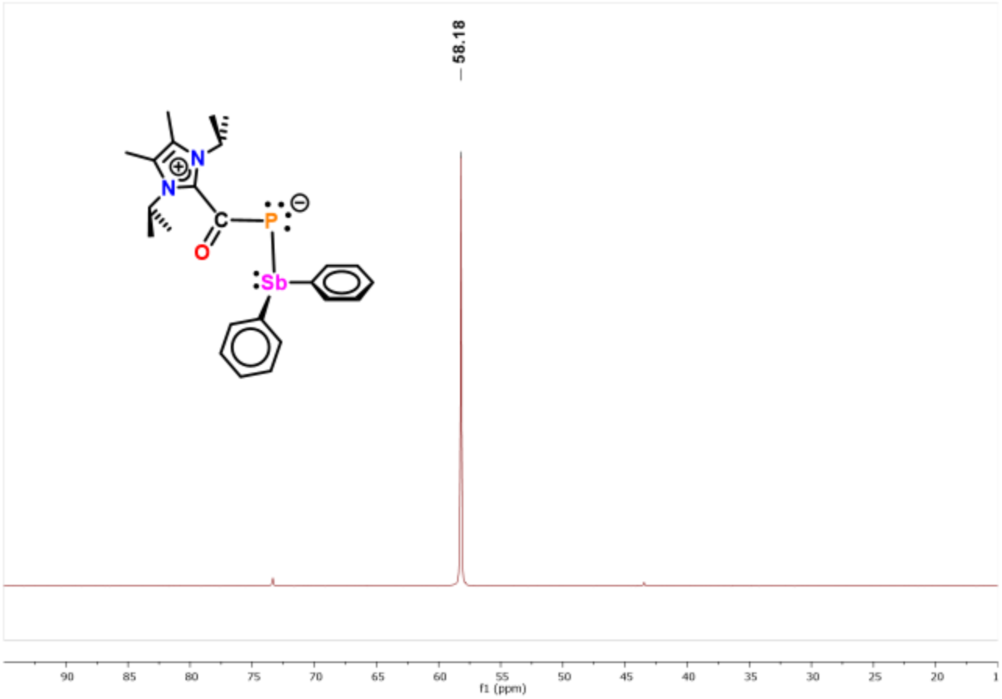

Figure S7: $^{31}P\{^{1}H\}$ NMR spectrum (202.46 MHz, 298 K) of complex **3**.

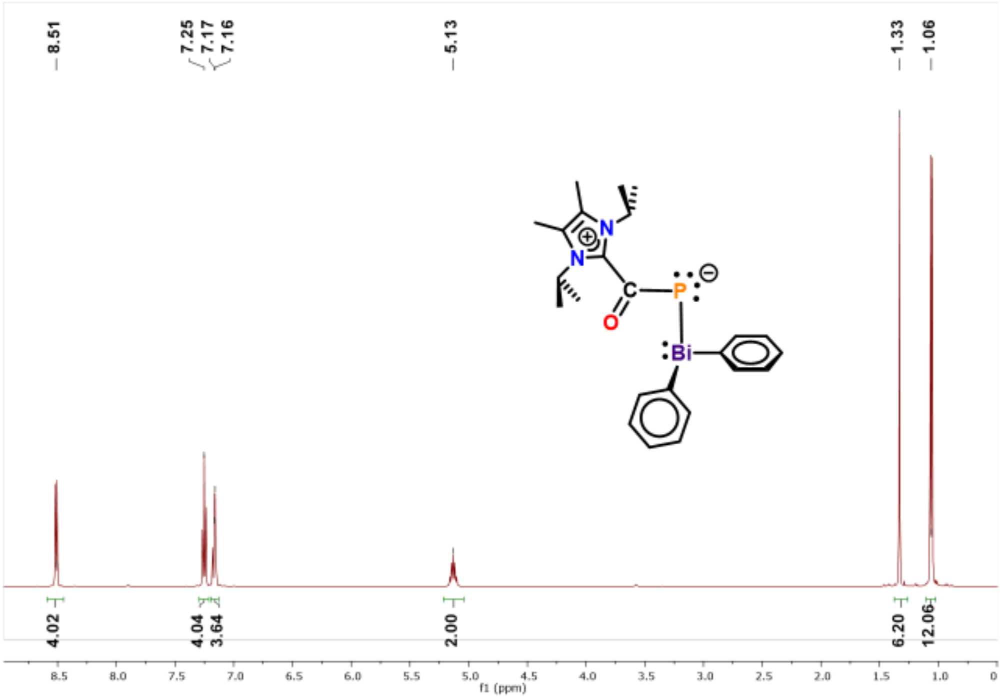

Figure S8: ^{1}H NMR spectrum (500.13 MHz, C_6D_6, 298 K) of complex **4**.

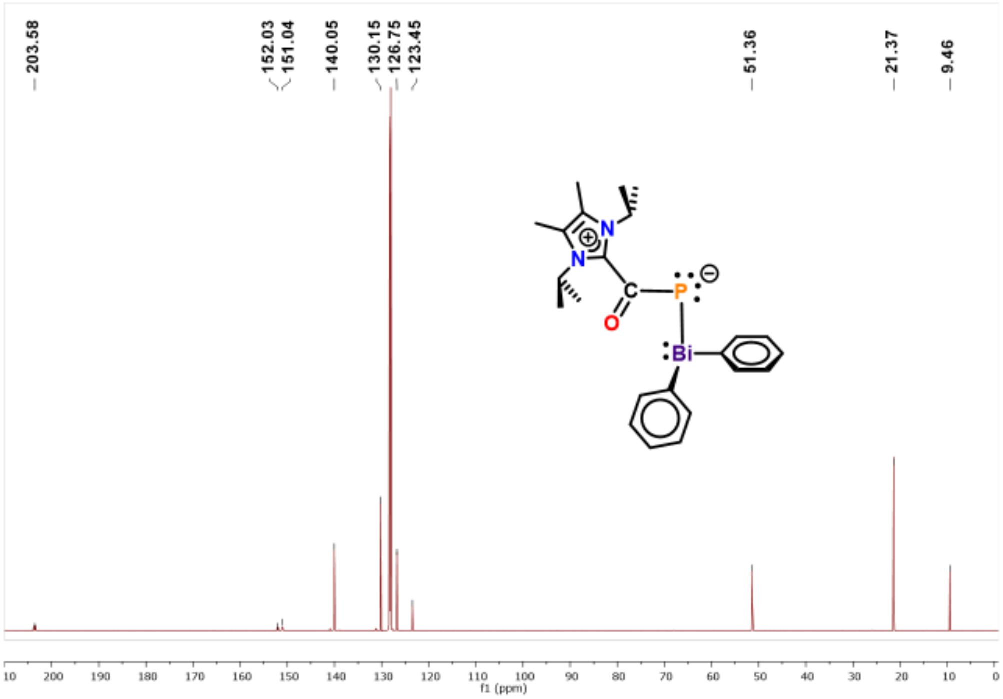

Figure S9: $^{13}C\{^{1}H\}$ NMR spectrum (201.193 MHz, C_6D_6, 298 K) of complex **4**.

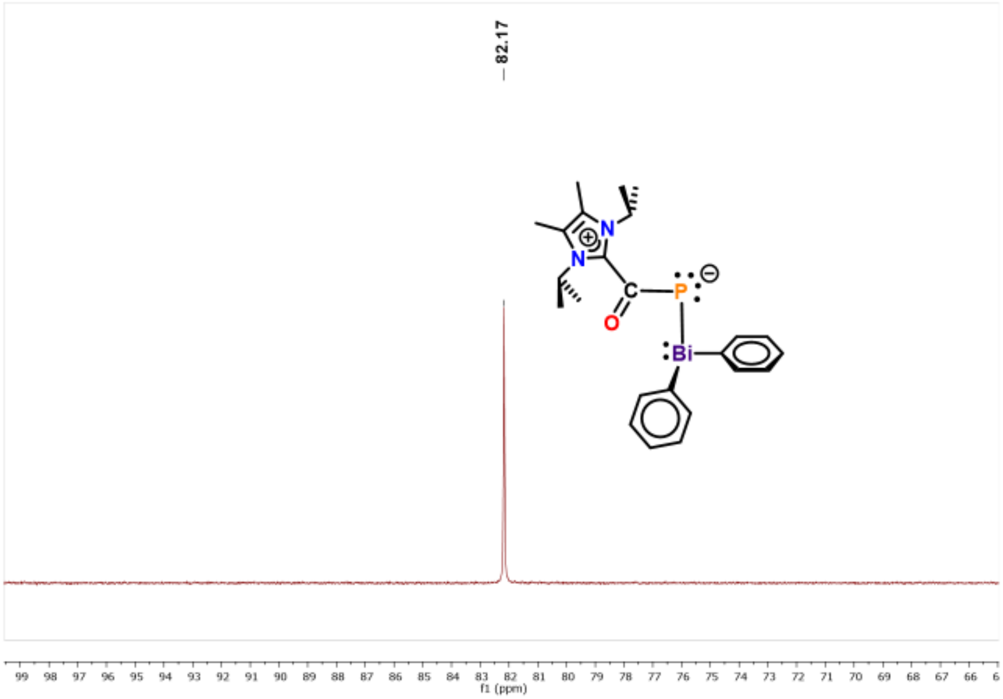

Figure S10: $^{31}P\{^{1}H\}$ NMR spectrum (202.46 MHz, 298 K) of complex **4**.

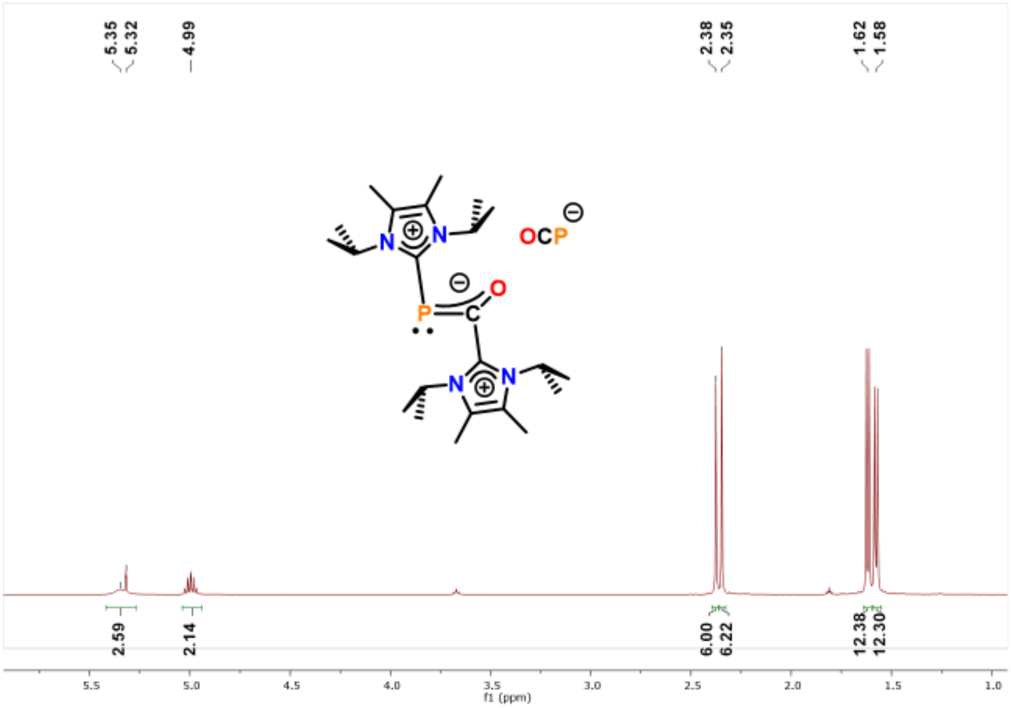

Figure S11: ^{1}H NMR spectrum (500.13 MHz, C_6D_6, 298 K) of complex **5**.

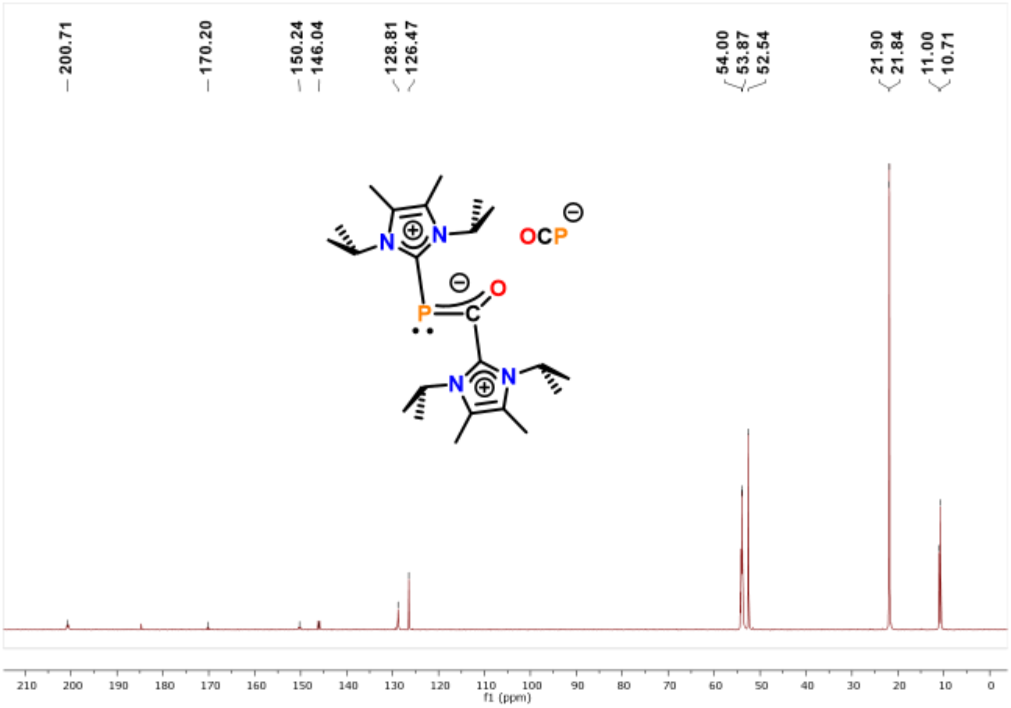

Figure S12: $^{13}C\{^{1}H\}$ NMR spectrum (201.193 MHz, C_6D_6, 298 K) of complex **5**.

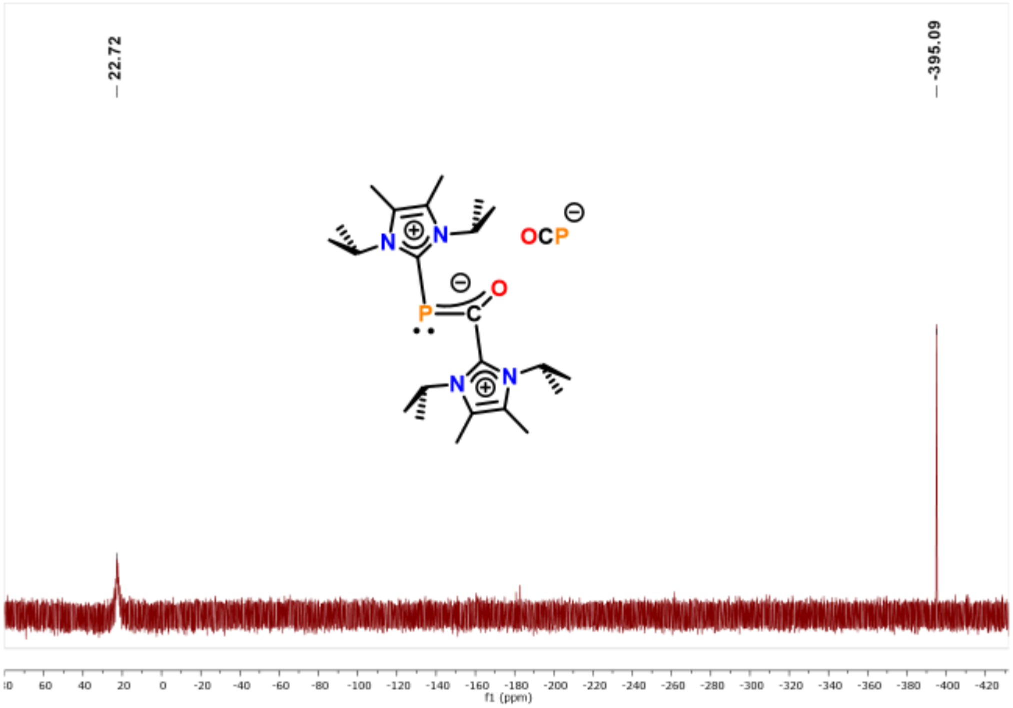

Figure S13: $^{31}P\{^{1}H\}$ NMR spectrum (242.94 MHz, 298 K) of complex **5**.

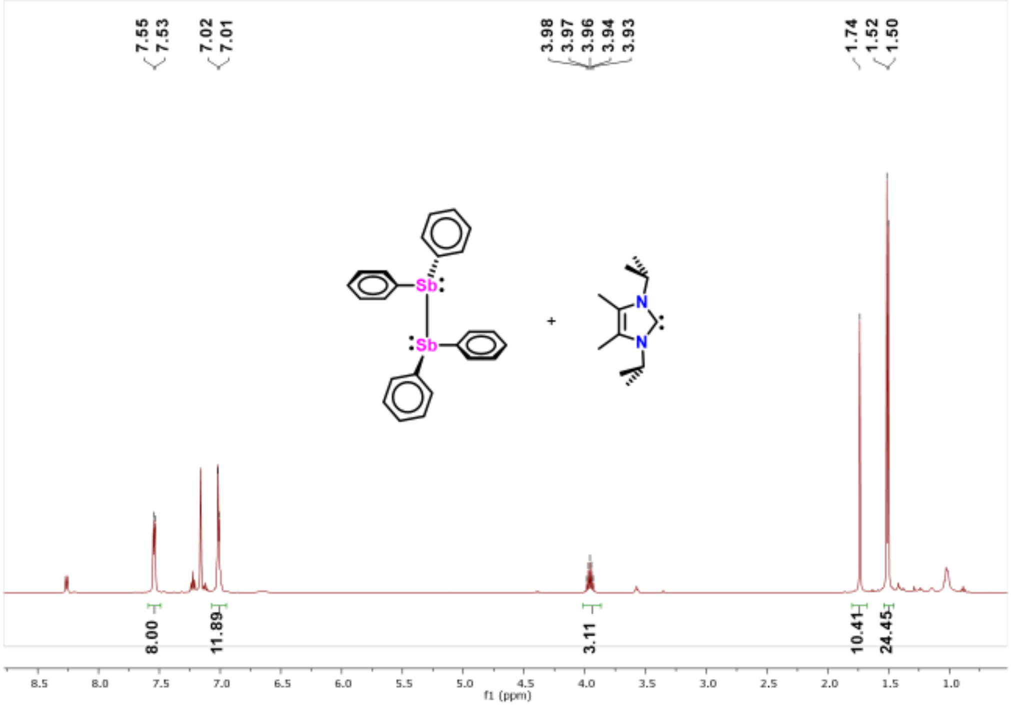

Figure S14: ^{1}H NMR spectrum (500.13 MHz, C_6D_6, 298 K) of the reaction mixture after heating compound **3** at 90 °C for 24 hours.

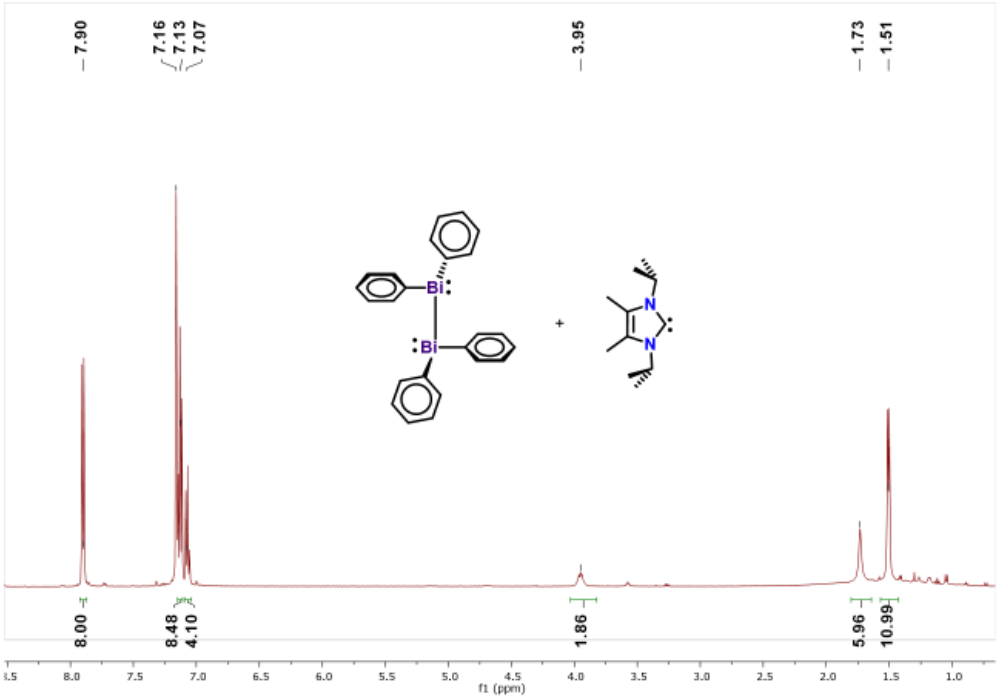

Figure S15: ^{1}H NMR spectrum (500.13 MHz, C_6D_6, 298 K) of the reaction mixture after heating compound **4** at 70 °C for 3 hours.

IR Spectral Data

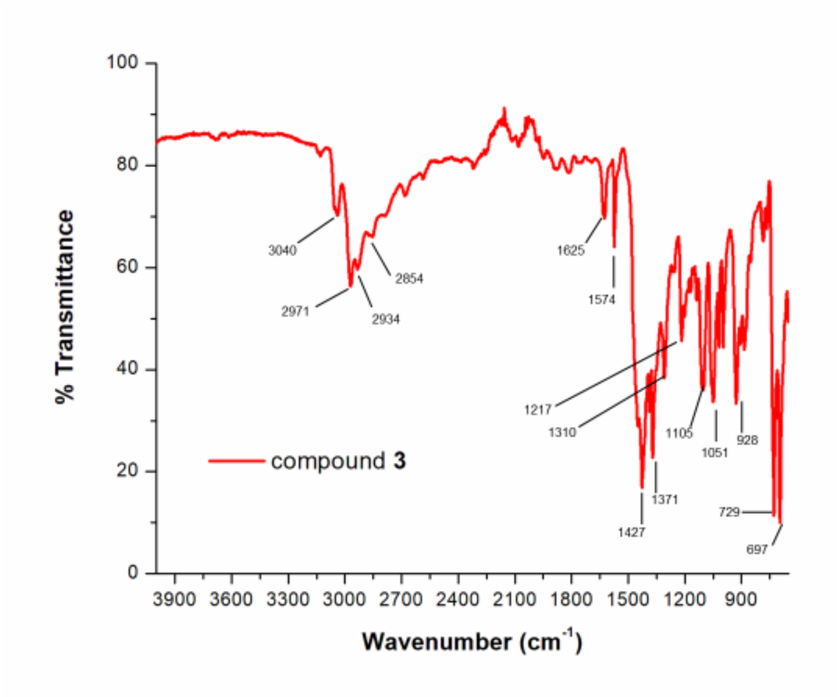

Figure S16: FT-IR Spectrum of compound **3**.

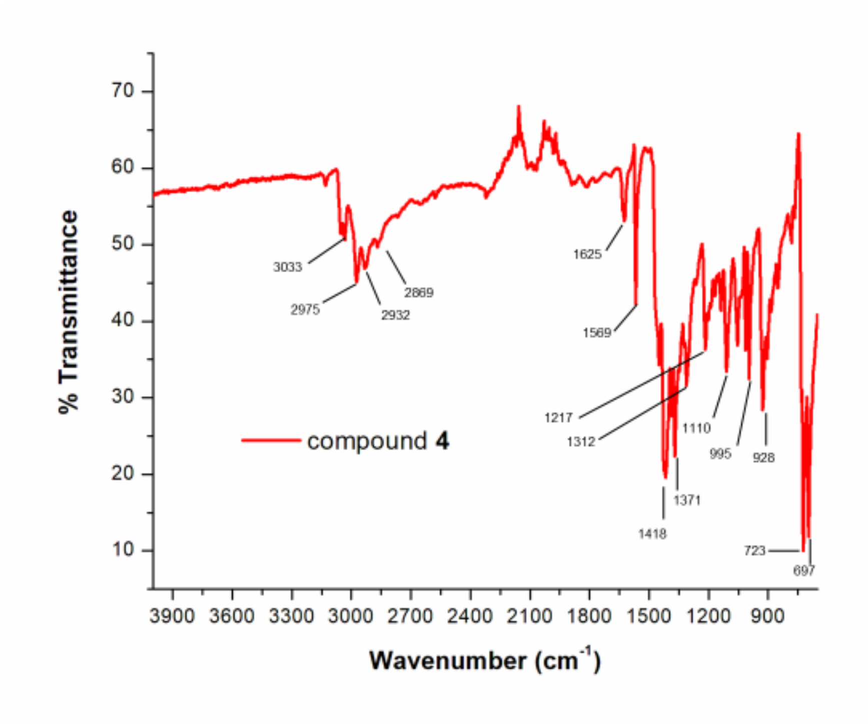

Figure S17: FT-IR Spectrum of compound **4**.

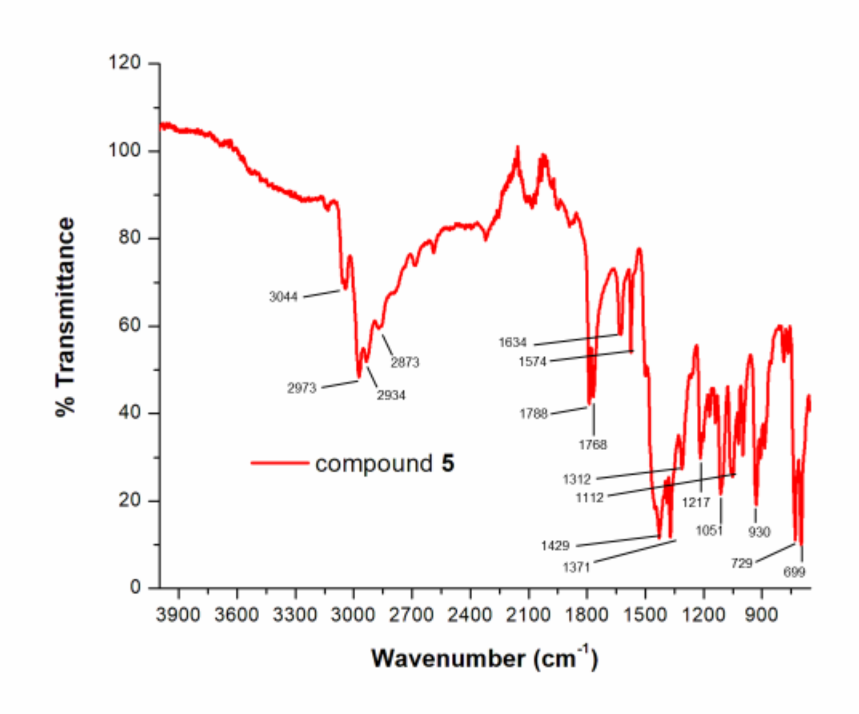

Figure S18: FT-IR Spectrum of compound **5**.

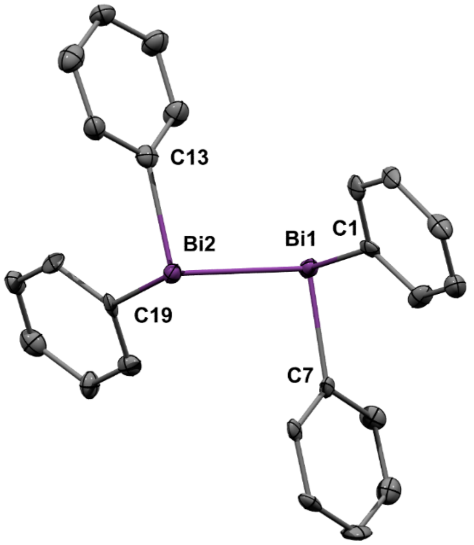

Figure S19: X-ray crystal structure of **6**. Ellipsoids represent 50% probability and all hydrogen atoms have been omitted for clarity. Selected bond lengths (Å) and angles (°): Bi1–Bi2: 2.9867(7); Bi1–C1: 2.274(13); Bi1–C7: 2.267(12); Bi2–C13: 2.261(13); Bi2–C19: 2.261(12). C1–Bi1–Bi2: 90.5(3); C7–Bi1–Bi2: 94.0(3); C7–Bi1–C1: 92.3(4); Bi1–Bi2–C13: 95.0(3); Bi1–Bi2–C19: 91.9(3); C13–Bi2–C19: 93.0(5).

Crystallographic Refinement Details

Table S1. Crystallographic data for **1-6**.

	**1**	**2**	**3**	**4**	**5**	**6**
**CCDC**	1962839	1962840	1962841	1962842	1976639	1962843
**Chemical formula**	$C_{23}H_{30}ClN_2Sb$	$C_{23}H_{30}BiClN_2$	$C_{24}H_{30}N_2OPSb$	$C_{24}H_{30}BiN_2OP$	$C_{24}H_{38}N_4O_2P_2$	$C_{24}H_{20}Bi_2$
**FW (g/mol)**	491.69	578.92	515.22	602.45	476.52	726.36
**T (K)**	100(2)	100(2)	100(2)	100(2)	100(2)	100(2)
**λ (Å)**	1.54178	0.71073	1.54178	1.54178	0.71073	0.71073
**Crystal size (mm)**	0.063 × 0.086 × 0.134	0.075 × 0.093 × 0.125	0.027 × 0.033 × 0.179	0.046 × 0.049 × 0.088	0.168 × 0.314 × 0.379	0.066 × 0.087 × 0.305
**Crystal habit**	colorless plate	colorless rod	yellow rod	yellow rod	orange block	yellow rod
**Crystal system**	triclinic	triclinic	triclinic	monoclinic	orthorhombic	monoclinic
**Space group**	P -1	P -1	P -1	P 2_1/c	F dd2	P2_1
**a (Å)**	9.9641(14)	9.9533(12)	11.3208(7)	11.2834(3)	17.553(2)	5.9018(6)
**b (Å)**	10.2681(16)	10.1962(12)	13.4788(9)	18.2355(5)	22.829(2)	21.746(2)
**c (Å)**	10.9350(14)	11.0990(14)	17.8244(12)	23.2146(6)	13.3554(15)	16.0512(16)
**α (°)**	90.719(10)	91.596(4)	92.540(4)	90	90	90
**β (°)**	96.231(9)	95.821(4)	108.009(4)	95.508(2)	90	92.145(3)
**γ (°)**	93.539(11)	93.214(4)	111.776(4)	90	90	90
**V (Å^3)**	1109.8(3)	1118.2(2)	2362.8(3)	4754.5(2)	5351.7(10)	2058.5(4)
**Z**	2	2	4	8	8	4
**ρ_{calc} (g/cm$^{3)}$**	1.471	1.719	1.448	1.683	1.183	2.344
**μ (mm^{-1})**	11.024	8.013	10.023	15.313	0.189	17.078
**F(000)**	500	564	1048	2352	2048	1320
**θ range (°)**	4.07 - 68.38°	3.69 - 51.48	2.65 - 68.53	3.09 - 68.39	2.11 to 25.35	2.53 - 56.70
**Index ranges**	$-11 \leq h \leq 11$<br>$-12 \leq k \leq 12$<br>$-13 \leq l \leq 12$	$-12 \leq h \leq 12$<br>$-12 \leq k \leq 12$<br>$-13 \leq l \leq 11$	$-13 \leq h \leq 13$<br>$-16 \leq k \leq 16$<br>$-19 \leq l \leq 21$	$-10 \leq h \leq 13$<br>$-21 \leq k \leq 21$<br>$-27 \leq l \leq 27$	$-20 \leq h \leq 21$<br>$-27 \leq k \leq 26$<br>$-15 \leq l \leq 16$	$-7 \leq h \leq 7$<br>$-28 \leq k \leq 28$<br>$-21 \leq l \leq 21$
**Reflns coll.**	14729	18501	34781	37832	10231	36083
**Ind. reflns**	4033 [R_{int} = 0.0593]	4264 [R_{int} = 0.0725]	8638 [R_{int} = 0.0681]	8710 [R_{int} = 0.0899]	2390 [R(int) = 0.0350]	10223 [R_{int} = 0.0667]
**Data / restraints / parameters**	4033 / 0 / 250	4264/0/250	8638 / 0 / 550	8710 / 0 / 559	2390 / 23 / 218	10223/1/469
**Goodness-of-fit on F^2**	1.053	1.034	1.057	1.012	1.079	1.009
**R_1 [I>2σ(I)]**	0.0338	0.0370	0.0604	0.0411	0.0519	0.0364
**wR_2 [all data]**	0.0875	0.0673	0.1643	0.0957	0.1414	0.0705

Computational Details

The computations were carried out with the Gaussian 09 suite of programs.[1] The structures were optimized using the ωB97XD functionals. We applied the def2-SVP basis set and we validated the calculations with def2-TZVP basis set implemented in Gaussian 09. At each of the optimized structures vibrational analysis was accomplished to check whether the stationary point located is a minimum or a saddle point of the potential energy hypersurface. We neglected the solvent-effect, because toluene was used as solvent, so the gas-phase computations simulated the reaction-conditions well. For Wiberg Bond Indexes and NPA charges the NBO program version 3.1 was employed.[2] The plotting of the orbitals was carried out with the AVOGADRO program (www.avogadro.cc).

Figure S20. Chemical structure of NHC-carbene with Cartesian X,Y,Z-coordinates (ωb97xd def2-TZVP).

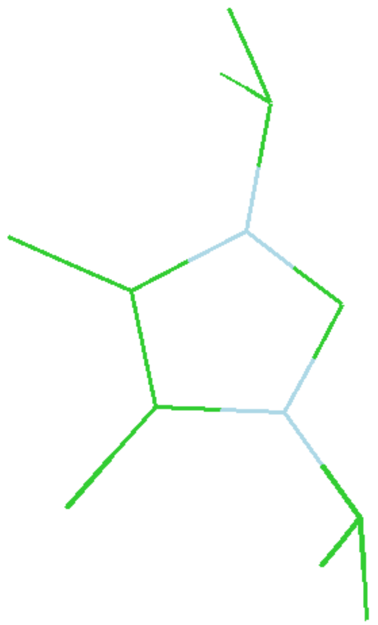

scf done: -540.723559

C	0.004640	-0.001670	0.004960
C	0.004648	0.002192	1.359943
N	1.342368	0.002239	1.744223
C	2.183472	-0.008090	0.682455
N	1.342313	-0.011877	-0.379312
C	-1.160982	0.028810	2.287777
C	1.890975	-0.055945	3.098484
C	1.406931	1.080295	3.991267
C	1.891290	0.042113	-1.733590
C	1.398154	-1.090207	-2.626367
C	-1.161127	-0.019517	-0.922910
C	1.691918	-1.428165	3.731768
C	1.702969	1.415902	-2.366757
H	2.958374	0.081466	2.927466
H	2.957591	-0.103706	-1.562678
H	-2.078915	0.180943	-0.372385
H	-1.079079	0.740598	-1.700236
H	-1.276183	-0.986920	-1.415938
H	-1.268784	0.997054	2.780785
H	-2.080230	-0.164760	1.737223
H	-1.084669	-0.731895	3.065111
H	2.012469	-1.132842	-3.527101
H	1.478231	-2.049061	-2.113131
H	0.364558	-0.948352	-2.942937
H	2.239471	1.464008	-3.315966
H	0.652862	1.633145	-2.568407
H	2.095505	2.190177	-1.707357

H	2.228094	-1.480410	4.680943
H	0.640148	-1.637128	3.933509
H	2.078310	-2.205549	3.072397
H	2.021857	1.118265	4.891794
H	1.494305	2.038435	3.477886
H	0.372383	0.946542	4.308227

Figure S21. Chemical structure of $BiPh_2PCO$ with Cartesian X,Y,Z-coordinates (ωb97xd def2-TZVP with contour value 0,05).

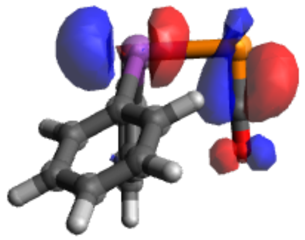

scf done: -1132.699499

C	0.178933	0.034519	-0.059512
C	0.084326	-0.111317	1.322381
C	1.249800	-0.245630	2.067207
C	2.489362	-0.225693	1.442317
C	2.574975	-0.073909	0.067404
C	1.417052	0.056237	-0.684472
Bi	-1.966500	-0.214018	2.227461
C	-2.585568	1.815068	1.510055
C	-3.942077	2.128220	1.547999
C	-4.394364	3.361651	1.102892
C	-3.491346	4.292874	0.612711
C	-2.139272	3.989494	0.572876
C	-1.686702	2.756536	1.020318

P	-1.345979	0.381058	4.741081
H	-4.663535	1.412952	1.930364
H	-5.451233	3.595142	1.139115
H	-3.840973	5.256332	0.263527
H	-1.430604	4.716165	0.195225
H	-0.626336	2.538060	0.988784
H	1.199208	-0.355511	3.144630
H	3.390247	-0.324123	2.035175
H	3.542460	-0.052996	-0.418274
H	1.477160	0.178728	-1.758832
H	-0.713710	0.151040	-0.665901
C	-0.613785	1.767891	4.205453
O	-0.086683	2.742549	3.887146

Table S2. Bond length and Wiberg-bond index of characteristic bonds.

Bond	Bond lengths (Å)	Wiberg-bond index
Bi-P	2,657	0,76
P-C	1,657	1,72
C-O	1,153	1,93

Table S3. Natural charges of main atoms.

Atom	Charge
Bi	+1,01
P	-0,14
C	+0,31
O	-0,47

Figure S22. Chemical structure of $SbPh_2PCO$ with Cartesian X,Y,Z-coordinates (ωb97xd def2-TZVP with contour value 0,05).

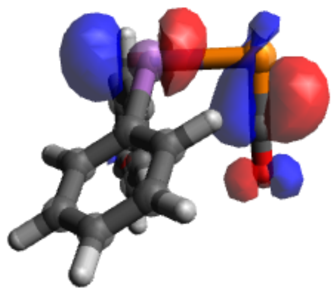

scf done: -1158.319675

C	0.193646	0.238025	-0.004771
C	0.084726	-0.027820	1.358975
C	1.233279	-0.352294	2.073471
C	2.467765	-0.397376	1.441994
C	2.567406	-0.121877	0.087293
C	1.427954	0.196457	-0.635619
Sb	-1.871697	-0.082962	2.249146
C	-2.548762	1.818484	1.519423
C	-3.925804	2.028303	1.483993
C	-4.447589	3.228215	1.025439
C	-3.595026	4.231699	0.591901
C	-2.223014	4.032668	0.621781
C	-1.701207	2.833112	1.082503

P	-1.299823	0.503007	4.675085
H	-4.606698	1.251802	1.816903
H	-5.519928	3.376755	1.003091
H	-3.999388	5.168941	0.230606
H	-1.553647	4.815149	0.286603
H	-0.627220	2.694879	1.099557
H	1.173738	-0.561292	3.135514
H	3.353614	-0.646825	2.012596
H	3.531622	-0.152443	-0.404294
H	1.498569	0.415679	-1.693793
H	-0.684492	0.496566	-0.586690
C	-0.452451	1.828004	4.143145
O	0.149280	2.756460	3.823278

Table S4. Bond length and Wiberg-bond index of characteristic bonds.

Bond	Bond lengths (Å)	Wiberg-bond index
Sb-P	2,551	0,79
P-C	1,662	1,70
C-O	1,152	1,94

Table S5. Natural charges of main atoms.

Atom	Charge
Sb	+0,97
P	-0,13
C	+0,32
O	-0,46

Figure S23. Chemical structure of NHC-$BiPh_2PCO$ complex with carthesian X,Y,Z-coordinates (ωb97xd def2-TZVP).

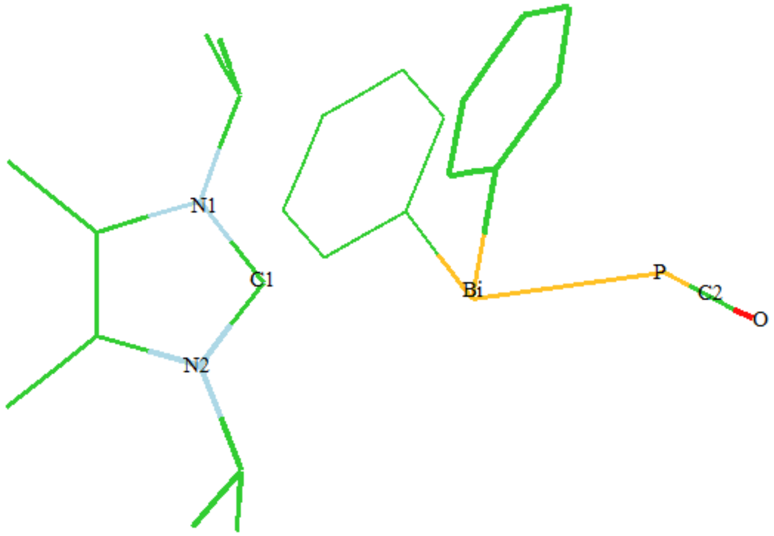

scf done: -1673.457288

N	2.182473	0.937557	-0.565611
C	1.576450	-0.266264	-0.574962
N	2.590499	-1.157322	-0.549933
C	3.825456	-0.520790	-0.507149
C	3.563509	0.809505	-0.521955
C	2.326610	-2.599586	-0.542490
C	2.553637	-3.211636	0.833441
C	5.149227	-1.203330	-0.474701
C	4.528514	1.942728	-0.472558
C	1.403970	2.179281	-0.676816
C	1.521830	2.784536	-2.069252
C	3.060196	-3.343054	-1.651566
C	1.702773	3.177986	0.431593
Bi	-1.020488	-0.897046	0.264061
P	-3.513234	-0.890805	1.504627

C	-4.108282	-1.534845	0.115626
O	-4.548537	-1.987687	-0.859959
C	-1.663234	0.519447	-1.371505
C	-1.066932	0.381312	-2.621475
C	-1.446876	1.187232	-3.684817
C	-2.423988	2.154316	-3.506657
C	-3.030146	2.295177	-2.268039
C	-2.660921	1.473747	-1.211393
C	-0.381183	0.575578	1.853997
C	0.767682	0.285553	2.583768
C	1.187883	1.112894	3.615805
C	0.464577	2.254704	3.925131
C	-0.677769	2.558576	3.200464
C	-1.099793	1.720612	2.177121
H	0.376798	1.847465	-0.550920
H	1.261274	-2.664819	-0.760852
H	5.925344	-0.487060	-0.211944
H	5.182285	-2.000431	0.267596
H	5.410112	-1.636354	-1.441907
H	4.514355	2.439995	0.498514
H	5.538922	1.577927	-0.647522
H	4.316550	2.692776	-1.233566
H	2.619365	-4.333835	-1.767100
H	2.963441	-2.816261	-2.601407
H	4.118586	-3.479430	-1.432786
H	2.273041	-4.265586	0.819258
H	3.597537	-3.146764	1.142242

H	1.942206	-2.708150	1.583087
H	0.791553	3.587797	-2.174809
H	2.512078	3.203817	-2.254457
H	1.306833	2.035725	-2.830519
H	0.911638	3.927886	0.448312
H	1.717884	2.687788	1.404351
H	2.647648	3.699107	0.277817
H	-3.181002	1.563511	-0.265084
H	-3.805417	3.038110	-2.124919
H	-2.720171	2.788740	-4.332846
H	-0.976554	1.060912	-4.652668
H	-0.282396	-0.352341	-2.771727
H	-2.006764	1.967137	1.638443
H	-1.248676	3.448415	3.436446
H	0.789351	2.905241	4.727535
H	2.081882	0.866180	4.175838
H	1.359671	-0.592805	2.347929

Table S6. Bond length and Wiberg-bond index of characteristic bonds.

Bond	Bond lengths (Å)	Wiberg-bond index
N1-C1	1,348	1,28
N2-C1	1,350	1,27
C1-Bi	2,801	0,26
C2-O	1,162	1,86
P-C2	1,643	1,84
Bi-P	2,784	0,54

Table S7. Natural charges of main atoms.

Atom or unit	Charge
N1	-0,37
N2	-0,38
C1	+0,14
C2	+0,26
O	-0,51
P	-0,23
Bi	+1,12
Carbene	+0,19

Figure S24. Chemical structure of NHC-SbPh$_2$PCO complex with Cartesian X,Y,Z-coordinates (ωb97xd def2-TZVP).

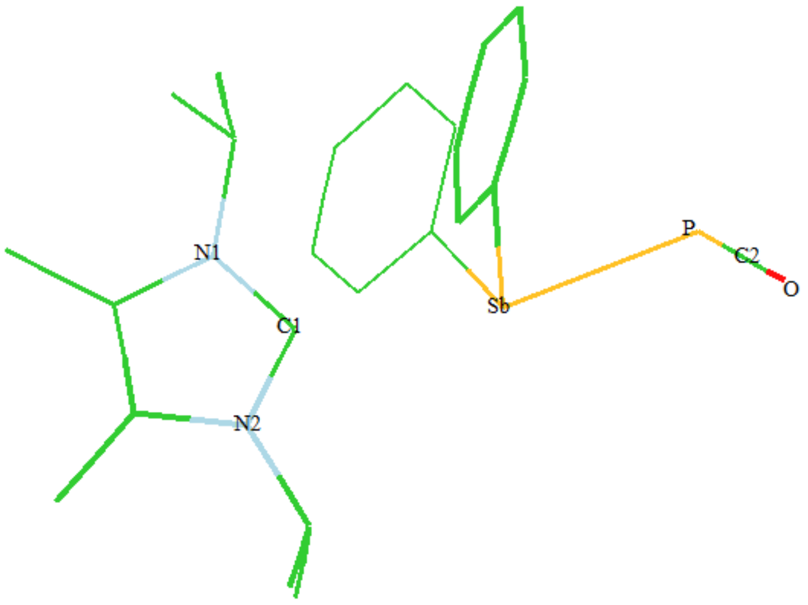

scf done: -1699.073067

C	1.535949	2.693747	0.189223
C	0.740867	1.891601	-0.622081
C	-0.331840	2.483595	-1.284227
C	-0.605079	3.836049	-1.142826
C	0.187398	4.619814	-0.317990

C	1.256607	4.043882	0.349880
Sb	1.100449	-0.210428	-1.006004
C	1.482640	-1.077587	0.939322
C	0.850604	-2.285872	1.220658
C	1.080054	-2.952613	2.414737
C	1.936571	-2.406169	3.358288
C	2.573321	-1.204453	3.090088
C	2.359577	-0.554957	1.881974
P	3.701586	0.471619	-1.370352
C	4.060778	-1.128255	-1.278349
O	4.325002	-2.257257	-1.212887
C	-1.381438	-0.352472	-0.122339
N	-1.958837	0.218983	0.952616
C	-3.333702	0.025411	0.946227
C	-3.615656	-0.694810	-0.166681
N	-2.399415	-0.920834	-0.800266
C	-1.161020	0.883293	1.994242
C	-1.577878	2.328460	2.223008
C	-2.172985	-1.628950	-2.065543
C	-2.760180	-3.034710	-2.074137
C	-4.939406	-1.185188	-0.642479
C	-4.283633	0.560359	1.961437
C	-2.605267	-0.792756	-3.262260
C	-1.101793	0.060023	3.273222
H	-0.159474	0.899763	1.577008
H	-1.090292	-1.731035	-2.115000
H	-5.736768	-0.664762	-0.115094

H	-5.086635	-1.004791	-1.706809
H	-5.066694	-2.254485	-0.464708
H	-4.435816	1.634330	1.841272
H	-5.250988	0.072656	1.856656
H	-3.939554	0.384368	2.979098
H	-2.324309	-3.594190	-2.902398
H	-2.523760	-3.559915	-1.147958
H	-3.841051	-3.036237	-2.210427
H	-2.337841	-1.307227	-4.186072
H	-3.681976	-0.618589	-3.275000
H	-2.100450	0.173789	-3.253888
H	-0.379175	0.511099	3.954332
H	-2.062441	0.016919	3.787787
H	-0.769165	-0.954986	3.059133
H	-0.808279	2.825711	2.814041
H	-1.663008	2.858660	1.274650
H	-2.518708	2.411700	2.767283
H	2.906616	0.354998	1.670213
H	3.255508	-0.778443	3.815634
H	2.114352	-2.919191	4.295131
H	0.589026	-3.898540	2.608224
H	0.159870	-2.716385	0.502884
H	2.396329	2.274225	0.695362
H	1.886411	4.649774	0.989675
H	-0.023400	5.675295	-0.200120
H	-1.439625	4.276961	-1.674391
H	-0.974579	1.882445	-1.918752

Table S8. Bond length and Wiberg-bond index of characteristic bonds.

Bond	Bond lengths (Å)	Wiberg-bond index
N1-C1	1,347	1,28
N2-C1	1,349	1,27
C1-Sb	2,638	0,32
C2-O	1,161	1,87
P-C2	1,642	1,82
Sb-P	2,714	0,55

Table S9. Natural charges of main atoms.

Atom or unit	Charge
N1	-0,38
N2	-0,37
C1	+0,17
C2	+0,27
O	-0,51
P	-0,23
Sb	+1,06
Carbene	+0,23

Figure S25. Chemical structure of NHC-C(O)PBiPh$_2$ complex with Cartesian X,Y,Z-coordinates (ωb97xd def2-TZVP).

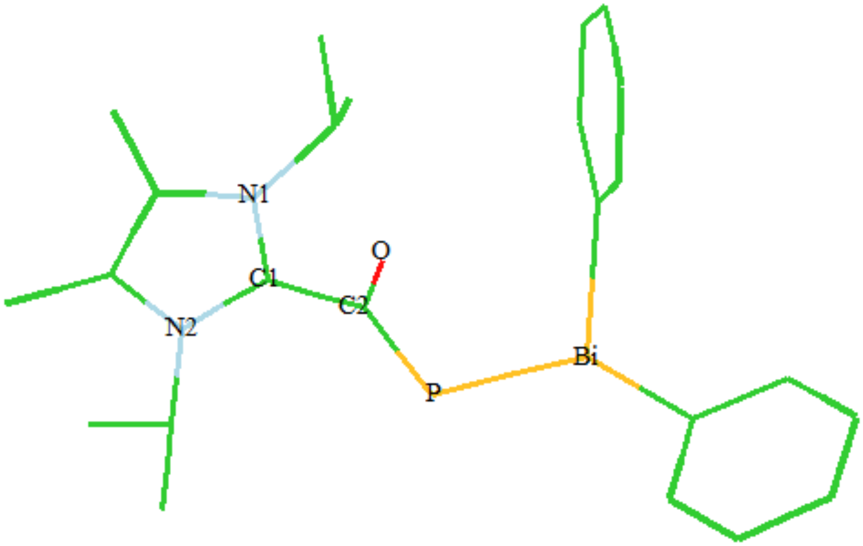

scf done: -1673.469611

C	3.260243	-1.908602	1.567785
C	3.383675	-0.921309	0.593476
C	4.527893	-0.125712	0.605012
C	5.515454	-0.303832	1.563709
C	5.377302	-1.291644	2.527371
C	4.247540	-2.094818	2.526444
Bi	1.837473	-0.629560	-1.036163
C	1.577474	1.568592	-0.572297
C	1.154886	2.431517	-1.579249
C	0.942629	3.779490	-1.320617
C	1.146809	4.283264	-0.044934
C	1.562749	3.431276	0.969022
C	1.773253	2.084679	0.706185
P	-0.154796	-1.289772	0.500521
C	-1.247919	-0.483447	-0.592019
O	-1.096070	0.034751	-1.714617
C	-2.620566	-0.261024	0.003036
N	-3.633972	-1.124702	0.063856
C	-4.729520	-0.487726	0.634798
C	-4.346413	0.786917	0.905269
N	-3.024164	0.905784	0.504192
C	-3.506768	-2.519784	-0.397946
C	-4.569823	-2.883832	-1.423973
C	-2.141940	2.093226	0.525178
C	-2.050291	2.720654	1.906315
C	-5.137016	1.880118	1.533299

C	-6.055851	-1.125045	0.857520
C	-2.531173	3.064896	-0.577034
C	-3.433006	-3.481795	0.778014
H	0.972809	2.051935	-2.578310
H	0.614849	4.436897	-2.117471
H	0.987442	5.335699	0.157239
H	1.727831	3.817811	1.968140
H	2.094630	1.430908	1.509070
H	2.378736	-2.540044	1.596650
H	4.129959	-2.865942	3.278624
H	6.145783	-1.432053	3.277648
H	6.393805	0.330808	1.558268
H	4.652865	0.659673	-0.133858
H	-2.537063	-2.538548	-0.894020
H	-1.154423	1.699726	0.287819
H	-6.196467	1.633519	1.505002
H	-4.853479	2.033364	2.575585
H	-5.007014	2.825242	1.008601
H	-5.962802	-2.100798	1.332025
H	-6.658174	-0.499786	1.513221
H	-6.602998	-1.255199	-0.077323
H	-1.809986	3.881456	-0.593000
H	-2.495032	2.562017	-1.543136
H	-3.526064	3.487746	-0.425237
H	-1.217426	3.423526	1.898791
H	-2.949716	3.269629	2.184134
H	-1.842423	1.962655	2.662253

H -4.278785 -3.814816 -1.910817

H -5.548479 -3.043525 -0.972601

H -4.652115 -2.113580 -2.191115

H -3.226282 -4.485251 0.404504

H -2.622857 -3.194687 1.448438

H -4.367673 -3.520265 1.339024

Table S10. Bond length and Wiberg-bond index of characteristic bonds.

Bond	Bond lengths (Å)	Wiberg-bond index
N1-C1	1,332	1,30
N2-C1	1,333	1,30
C1-C2	1,513	0,93
C2-O	1,246	1,47
P-C2	1,743	1,42
Bi-P	2,601	0,91

Table S11. Natural charges of main atoms.

Atom or unit	Charge
N1	-0,29
N2	-0,29
C1	+0,41
C2	+0,04
O	-0,68
P	-0,24
Bi	+0,94
Carbene	+0,75

Figure S26. Chemical structure of NHC-C(O)PSbPh$_2$ complex with Cartesian X,Y,Z-coordinates (ωb97xd def2-TZVP).

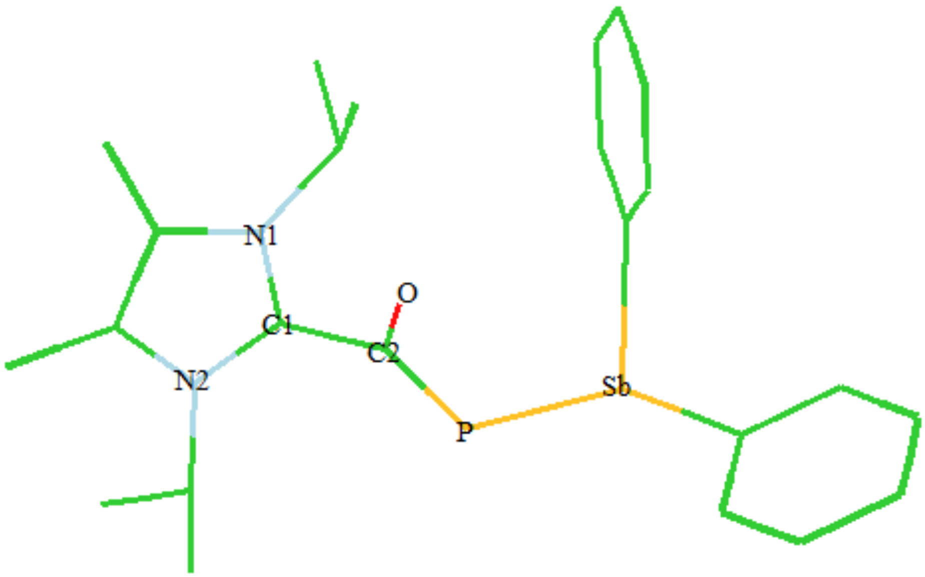

scf done: -1699.090604

N	-3.410275	-1.123139	-0.019538
C	-2.389142	-0.268841	-0.074650
N	-2.763583	0.880526	0.486062
C	-4.075218	0.759719	0.919726
C	-4.481236	-0.498599	0.608855
C	-1.037944	-0.477313	-0.723585
O	-0.916249	0.090641	-1.823122
C	-1.867377	2.057032	0.529138
C	-2.267412	3.069538	-0.531760
C	-4.834692	1.837447	1.609665
C	-5.806988	-1.131756	0.845933

C	-3.315553	-2.497742	-0.547057
C	-3.214664	-3.512429	0.581548
P	0.081786	-1.340085	0.298699
Sb	2.002378	-0.677720	-1.182969
C	1.829187	1.421599	-0.676097
C	1.420207	2.328060	-1.650123
C	1.239713	3.670719	-1.344189
C	1.466173	4.126233	-0.054356
C	1.872564	3.231893	0.926603
C	2.048525	1.891052	0.617535
C	3.530503	-1.082382	0.298215
C	4.726599	-0.367356	0.248390
C	5.751898	-0.621370	1.146850
C	5.602655	-1.608993	2.109523
C	4.423344	-2.334592	2.166873
C	3.397000	-2.071583	1.269423
C	-4.416951	-2.801068	-1.551850
C	-1.741931	2.638907	1.927519
H	1.223935	1.984044	-2.658882
H	0.919911	4.361346	-2.115461
H	1.331735	5.174551	0.184290
H	2.055857	3.580276	1.936415
H	2.359815	1.202575	1.394640
H	2.477334	-2.641276	1.343131
H	4.297175	-3.105681	2.917525
H	6.401163	-1.810464	2.812949
H	6.670357	-0.049124	1.093764

H	4.862072	0.412503	-0.494188
H	-2.363321	-2.505213	-1.076376
H	-0.889387	1.661136	0.259269
H	-5.895920	1.597190	1.614897
H	-4.510612	1.958600	2.644230
H	-4.718350	2.796537	1.107249
H	-5.709576	-2.127213	1.276760
H	-6.381953	-0.528831	1.545656
H	-6.384695	-1.217033	-0.075505
H	-1.537232	3.878308	-0.535555
H	-2.257597	2.598610	-1.514370
H	-3.253957	3.497928	-0.344646
H	-0.898699	3.329417	1.926587
H	-2.628244	3.191794	2.237871
H	-1.532385	1.855502	2.656662
H	-4.164754	-3.722722	-2.076666
H	-5.385992	-2.949592	-1.076410
H	-4.500986	-2.004216	-2.291138
H	-3.036198	-4.500596	0.156506
H	-2.376977	-3.266105	1.234177
H	-4.129165	-3.563151	1.174084

Table S12. Bond length and Wiberg-bond index of characteristic bonds.

Bond	Bond lengths (Å)	Wiberg-bond index
N1-C1	1,333	1,30
N2-C1	1,333	1,30
C1-C2	1,513	0,93
C2-O	1,244	1,48
P-C2	1,745	1,41
Sb-P	2,515	0,92

Table S13. Natural charges of main atoms.

Atom or unit	Charge
N1	-0,29
N2	-0,29
C1	+0,41
C2	+0,06
O	-0,67
P	-0,24
Sb	+0,90
Carbene	+0,76

References

1. M. J. Frisch, G. W. T., H. B. Schlegel, G. E. Scuseria, M. A. Robb, J. R. Cheeseman, G. Scalmani, V. Barone, B. Mennucci, G. A. Petersson, H. Nakatsuji, M. Caricato, X. Li, H. P. Hratchian, A. F. Izmaylov, J. Bloino, G. Zheng, J. L. Sonnenberg, M. Hada, M. Ehara, K. Toyota, R. Fukuda, J. Hasegawa, M. Ishida, T. Nakajima, Y. Honda, O. Kitao, H. Nakai, T. Vreven, J. A. Montgomery, Jr., J. E. Peralta, F. Ogliaro, M. Bearpark, J. J. Heyd, E. Brothers, K. N. Kudin, V. N. Staroverov, R. Kobayashi, J. Normand, K. Raghavachari, A. Rendell, J. C. Burant, S. S. Iyengar, J. Tomasi, M. Cossi, N. Rega, J. M. Millam, M. Klene, J. E. Knox, J. B. Cross, V. Bakken, C. Adamo, J. Jaramillo, R. Gomperts, R. E. Stratmann, O. Yazyev, A. J. Austin, R. Cammi, C. Pomelli, J. W. Ochterski, R. L. Martin, K. Morokuma, V. G. Zakrzewski, G. A. Voth, P. Salvador, J. J. Dannenberg, S. Dapprich, A. D. Daniels, Ö. Farkas, J. B. Foresman, J. V. Ortiz, J. Cioslowski and D. J. Fox, Gaussian 09, Revision A.02, Gaussian 09, Revision A.02. *Gaussian Inc. , Wallingford CT* **2009.**

2. Glendening, E. D.; Reed, A. E.; Carpenter, J. E.; Bohmann, J. A.; Morales, C. M.; Weinhold, F., In NBO 5.0., Theoretical Chemistry Institute, University of Wisconsin, Madison, 2001.
